# Tracing priming effects in palsa peat carbon dynamics using a stable isotope-assisted metabolomics approach

**DOI:** 10.3389/fmolb.2025.1621357

**Published:** 2025-08-22

**Authors:** Christian Ayala-Ortiz, Moira Hough, Elizabeth K. Eder, David W. Hoyt, Rosalie K. Chu, Jason Toyoda, Steven J. Blazewicz, Patrick M. Crill, Ruth Varner, Scott R. Saleska, Virginia I. Rich, Malak M. Tfaily

**Affiliations:** ^1^ Department of Environmental Science, University of Arizona, Tucson, AZ, United States; ^2^ College of Forest Resources and Environmental Sciences, Michigan Technological University, Houghton, MI, United States; ^3^ Environmental Molecular Sciences Laboratory, Pacific Northwest National Laboratory, Richland, WA, United States; ^4^ Physical and Life Sciences Directorate, Lawrence Livermore National Laboratory, Livermore, CA, United States; ^5^ Department of Geological Sciences and Bolin Centre for Climate Research, Stockholm University, Stockholm, Sweden; ^6^ Department of Earth Sciences and Earth Systems Research Center, University of New Hampshire, Durham, NH, United States; ^7^ Department of Ecology and Evolutionary Biology, University of Arizona, Tucson, AZ, United States; ^8^ Department of Microbiology, The Ohio State University, Columbus, OH, United States; ^9^ Center of Microbiome Science, The Ohio State University, Columbus, OH, United States; ^10^ Bio5 Institute, University of Arizona, Tucson, AZ, United States

**Keywords:** isotopic tracer, high resolution mass spectrometry, metabolomics, NMR, palsa, carbon cycling, stable isotope-assisted metabolomics, litter decomposition

## Abstract

**Introduction:**

Peatlands store up to a third of global soil carbon, and in high latitudes their litter inputs are increasing and changing in composition under climate change. Although litter significantly influences peatland carbon and nutrient dynamics by changing the overall lability of peatland organic matter, the physicochemical mechanisms of this impact—and thus its full scope—remain poorly understood.

**Methods:**

We applied multimodal metabolomics (UPLC-HRMS, ^1^H NMR) paired with ^13^C Stable Isotope-Assisted Metabolomics (SIAM) to track litter carbon and its potential priming effects on both existing soil organic matter and carbon gas emissions. Through this approach, we achieved molecule-specific tracking of carbon transformations at unprecedented detail.

**Results:**

Our analysis revealed several key findings about carbon dynamics in palsa peat. Microbes responded rapidly to litter addition, producing a short-term increase in CO_2_ emissions, fueled nearly exclusively by transformations of litter carbon. Litter inputs significantly contributed to the organic nitrogen pool through amino acids and peptide derivatives, which served as readily accessible nutrient sources for microbial communities. We traced the fate of plant-derived polyphenols including flavonoids like rutin, finding evidence of their degradation through heterocyclic C-ring fission, while accumulation of some polyphenols suggested their role in limiting overall decomposition. The SIAM approach detected subtle molecular changes indicating minimal and transient priming activity that was undetectable through conventional gas measurements alone. This transient response was characterized by brief microbial stimulation followed by rapid return to baseline metabolism. Pre-existing peat organic matter remained relatively stable; significant priming of its consumption was not observed, nor was its structural alteration.

**Discussion:**

This suggests that while litter inputs temporarily increase CO_2_ emissions, they don’t sustain long-term acceleration of stored carbon decomposition or substantially decrease peat’s carbon store capacity. Our findings demonstrate how technological advancements in analytical tools can provide a more detailed view of carbon cycling processes in complex soil systems.

## 1 Introduction

Metabolites are molecules that constitute the substrates and end products of cellular metabolism and regulatory processes (Fiehn, 2002). Metabolomics involves measuring thousands of naturally occurring metabolites within a biological system (collectively known as the metabolome) to provide a qualitative or quantitative analysis of its metabolic processes (Liu and Locasale, 2017). Unlike previous metabolite-based studies, current metabolomics tools, such as liquid chromatography coupled with tandem mass spectrometry and nuclear magnetic resonance (NMR) spectroscopy offer powerful approaches for comprehensive and rapid profiling of the entire suite of compounds derived from complex mixtures such as peat (Kellogg and Kang, 2020). These techniques facilitate the identification of organic compounds, decomposition byproducts, and elucidation of underlying metabolic pathways (Ristok et al., 2017; Fudyma et al., 2019; 2021; Tfaily et al., 2019; AminiTabrizi et al., 2020; AminiTabrizi et al., 2023; Wilson et al., 2021). Stable isotope probing has been successfully combined with ultrahigh-performance chromatography–tandem mass spectrometry to quantitate trace levels of ^13^C-labelled nucleic acids (Wilhelm et al., 2014), and when integrated with metabolomics, known as stable-isotope assisted metabolomics (SIAM), it can provide detailed characterization of the chemical species produced during metabolic processes (Wei et al., 2017; Wilhelm et al., 2022) allowing researchers to identify potential degradation products of labeled metabolites and track their fate along different metabolic pathways or environmental compartments (Tian et al., 2018; Hou et al., 2021).

These advanced analytical techniques are particularly valuable for studying critical ecosystems such as peatlands, which are vast carbon (C) reservoirs holding up to one-third of the terrestrial organic carbon (Lal, 2008; Mitsch et al., 2013; Dargie et al., 2017), and maybe even more as more current analysis estimated the amount of carbon in peatlands to be around of 1,055 Pg (Nichols and Peteet, 2019). Peatland ecosystems, especially those from higher latitudes including permafrost-affected peatlands, play a critical role in regulating atmospheric greenhouse gasses, particularly carbon dioxide (CO_2_) and methane (CH_4_) (Gorham, 1991; Lal, 2010; Nichols and Peteet, 2019). However, Arctic warming, which is occurring at a rate up to three times faster than the rest of the globe (Rantanen et al., 2022), alongside altered precipitation patterns are accelerating soil organic matter (SOM) decomposition, potentially transforming peatlands from carbon sinks to sources (Lunt et al., 2019; Smith et al., 2019; Qiu et al., 2020).

Accumulation of organic matter in high latitude peatlands results from an imbalance between plant inputs to the peat and their release as CO_2_ or dissolved organic carbon after decomposition (Moore and Basiliko, 2006). In these habitats, organic matter decomposition occurs slowly and depends on the interplay between different biotic and abiotic factors (Moore and Basiliko, 2006), with plant litter inputs playing a crucial role in determining the overall decomposition rates (Mastný et al., 2018; Wilson et al., 2022). Plant-derived materials significantly impact the degradability of peatland organic matter either by enriching the peat with readily available, low-molecular-weight compounds (Mastný et al., 2018), or by making it more resistant to degradation by releasing recalcitrant (less bioavailable) or microbial growth-inhibiting metabolites (Painter, 1991; Stalheim et al., 2009; Fudyma et al., 2019). Consequently, litter quality, determined by vegetation species, exerts direct influence on litter decomposition, especially over shorter time scales (Ward et al., 2015). For example, a previous study at Stordalen Mire, Sweden, using Fourier Transform Infrared Spectroscopy (FT-IR) and Fourier Transform Ion Cyclotron Resonance Mass Spectrometry (FTICR-MS), demonstrated that differences in litter composition and environmental factors among the permafrost thaw-sequence habitats of palsa, bog and fen influenced the mechanisms of litter degradation and the bioavailability of the metabolites that remain in the peat (Wilson et al., 2022). The chemical diversity of litter dictates the metabolic processes available to microbial communities for its degradation (Bhatnagar et al., 2018; AminiTabrizi et al., 2020). This chemical composition also influences the composition of these microbial communities themselves (Hättenschwiler and Vitousek, 2000; Wardle et al., 2006; Fofana et al., 2022). This effect is strongest at the beginning of the decomposition process when labile substrates and components of those substrates are available, and diminishes over time, allowing live vegetation to play a significant role (Ward et al., 2015).

As a result of climate warming there is a change in permafrost-affected peatland vegetation dynamics, favoring the spread of vascular plants over peat mosses, resulting in an increase of biomass due to higher productivity (Hodgkins et al., 2014; Buttler et al., 2015; Dieleman et al., 2015; Ward et al., 2015; Bell et al., 2018; Mastný et al., 2018; Norby et al., 2019; Wilson et al., 2021; Antala et al., 2022). This shift can modify current decomposition rates, as vascular plant litter typically contains higher nutrient content and is easier to degrade (Kaštovská et al., 2018) compared to *Sphagnum* spp. litter. *Sphagnum* spp. mosses, which dominate boreal and permafrost-affected ecosystems, limit decomposition through various mechanisms including acidification of its surroundings, accumulation of phenolic compounds, and production of antimicrobial substances (van Breemen, 1995; Chiapusio et al., 2018; Fudyma et al., 2019; Cory et al., 2025). Understanding these litter-driven processes requires advanced analytical approaches to track specific molecular transformations.

Beyond direct effects on decomposition rates, litter inputs also influence carbon cycling through more complex mechanisms. Litter inputs play a crucial role in determining the manifestation of priming effects, a key mechanism influencing carbon storage stability in peatlands (Liang et al., 2018). Priming refers to changes in decomposition rates of pre-existing SOM that occur due to the addition of an organic amendment to the soil (Bingeman et al., 1953; Liu et al., 2020). These inputs can result in the enhanced degradation of pre-existing SOM, known as “positive priming” (Kuzyakov et al., 2000), or in its reduction, known as “negative priming” (Guenet et al., 2010). Understanding priming effects is particularly important for predicting how changing litter inputs might affect the stability of stored carbon in peatlands. The exact mechanisms behind priming are still not completely understood, but primarily involve two key processes: microbial activation and carbon-nitrogen stoichiometry effects that emerge particularly under nitrogen limitation (Kuzyakov et al., 2000; Perveen et al., 2019; Wild et al., 2019; Liu et al., 2020). Microbial activation involves an increased microbial activity and growth supported by the additional substrate which allows microbial communities to then shift to native SOM sources (Kuzyakov et al., 2000; Liu et al., 2017), while the C-N stoichiometry model involves accessing the N contained in the SOM to meet the demands created by the additional carbon entering into the system (Kuzyakov et al., 2000; Fontaine et al., 2003).

Previous research at Stordalen Mire in Arctic Sweden showed that litter addition to active layer palsa peat produced a significant but transient spike in CO_2_ emissions that rapidly declined after 7 days (Hough, 2020). Although isotopic measurements indicated potential positive priming of SOM decomposition, substantial data variability prevented definitive conclusions. This critical knowledge gap persists because conventional methods lack the molecular resolution needed to identify and track the specific biochemical transformations occurring during early-stage litter decomposition. The underlying mechanisms driving priming effects in active layer palsa peat, including the precise metabolic pathways and molecular transformation sequences, remain uncharacterized.

To overcome these methodological limitations and address these fundamental knowledge gaps, we employed an integrated multimodal metabolomics approach using ultra performance liquid chromatography coupled with high resolution mass spectrometry and ^1^H NMR (UPLC-HRMS, ^1^H NMR), paired with stable isotope labeling to precisely track carbon transformations and characterize how litter addition affects organic matter composition of active layer palsa peat at the molecular level. While previous environmental studies have successfully applied SIAM to gain insights into biotransformation of pollutants (Tian et al., 2018), or specific metabolites of interest (Chen et al., 2022; Qian et al., 2023), they typically trace only a single labeled compound. Our approach represents a significant methodological advancement—we labeled native plants *in vivo* to create a diverse pool of isotopically labeled compounds, then amended peat incubations with this complex labeled litter under field-matched conditions. This novel application of SIAM to soil-litter interactions enabled us to simultaneously track carbon through multiple transformation pathways and detect subtle priming effects that conventional methods would miss.

Our investigation had two primary objectives: (1) to elucidate specific litter decomposition pathways by tracing litter metabolites through their transformation products, and (2) to characterize impacts on carbon and nutrient cycling processes that ultimately influence climate-relevant carbon gas dynamics (Tian et al., 2018; Hou et al., 2021). We hypothesized that fresh litter addition would trigger an immediate metabolic response as microbial communities rapidly degraded the bioavailable and nutritious contents present in the litter inputs corresponding to an observed CO_2_ efflux peak. Our second hypothesis addressed priming effects. Despite the potential for labile litter decomposition to stimulate microbial activity, we expected the high C ratios documented in palsa litter (Hough et al., 2022) would limit strong positive priming effects—consistent with the rapid decline in CO_2_ release observed in previous studies (Hough et al., 2022).

Our integrated approach linking metabolomic profiles to previous respiration data aimed to develop a mechanistic understanding of how litter contributes to active layer palsa peat formation and respiration over the course of decomposition. This research enhances our understanding of how litter inputs modulate carbon and nutrient cycling processes in permafrost-affected peatlands, ultimately leading to changes in climate forcing gas dynamics and climate feedback. Our findings have important implications for improving climate change predictions and informing peatland management strategies in response to ongoing environmental changes.

## 2 Materials and methods

### 2.1 Study site

The Stordalen Mire (68°21′N 18°49′E) is a peat plateau located adjacent to Lake Torneträsk, underlain by discontinuous permafrost in northernmost Sweden, 10 km east of Abisko. Ongoing monitoring and sampling have been conducted at the Stordalen Mire for decades, most recently focusing on plant and microbial community composition, soil organic matter chemistry, and CH_4_ and CO_2_ fluxes and isotopic ratios from the active layer zone (Malmer et al., 2005; Hodgkins et al., 2014; McCalley et al., 2014; Mondav et al., 2017; Holmes et al., 2022). The non-lake surface of this study site (∼98%) is covered by the three most commonly occurring subhabitats of northern wetlands (Johansson et al., 2006). These subhabitats include semi-drained palsas containing mixed low shrubs, mosses, lichen, and sedges and a shallow persistent active layer underlain by permafrost, semi-thawed bogs with variable seasonal water table and active layer depth with a dominant cover of *Sphagnum* spp. Mosses and small sedges, and fully thawed fens dominated by large sedges and without a discernible permafrost layer (Johansson et al., 2006; Hough et al., 2022).

Active layer peat soil and live plants for this study were collected from palsa sites which are raised, semi-drained ombrotrophic peat areas with underlying permafrost (Johansson et al., 2006; Olefeldt and Roulet, 2012). The surfaces are mostly dominated by a mixture of ericoid, graminoid, moss and lichen species (Hough et al., 2022; Wilson et al., 2022) (more details discussed below).

### 2.2 Plant labeling

In addition to its ecological relevance, *E. vaginatum* was chosen for practical reasons: it was the only species for which we could reliably obtain thoroughly isotopically labeled material. Its rapid growth rate and annual replacement of leaves facilitated effective ^13^C enrichment by growing living plants in a chamber with ^13^C-enriched CO_2_ at atmospheric concentrations. Living plants representative of the palsa vegetation (*E. vaginatum*) were isotopically labeled by growing them in a chamber with ^13^C-enriched CO_2_ at atmospheric concentrations of CO_2_ to obtain system-specific ^13^C labeled litter according to (Hough, 2020). Control plants were grown in a different chamber at the same CO_2_ concentration with naturally occurring levels of ^13^CO_2_. Briefly, living plants for labeling were gathered from the field at the start of the Arctic growing season (20 June 2016). Plants were collected intact to ensure they kept their root systems, and the soil matrix attached to them (Hough, 2020). The labeling period consisted of 8 weeks of growth from June 25 through August 11. During the experiment, the chamber air was regulated over the entire growing period to ensure proper atmospheric ^13^C enrichment at roughly 400 ppm CO_2_. All new plant growth (i.e., new leaves) was harvested after 8 weeks. These samples were collected, dried to constant weight, and ground. The ground material was then used for isotopic enrichment analysis at the Dept of Geological Sciences, Stockholm University Stable Isotope Laboratory, and subsequently utilized in the incubation experiments described below. Final enrichment of the plant material from *E. vaginatum* prior to addition to incubations was 52.4 atom percent (at-%).

### 2.3 Litter decomposition

**FIGURE 1:**
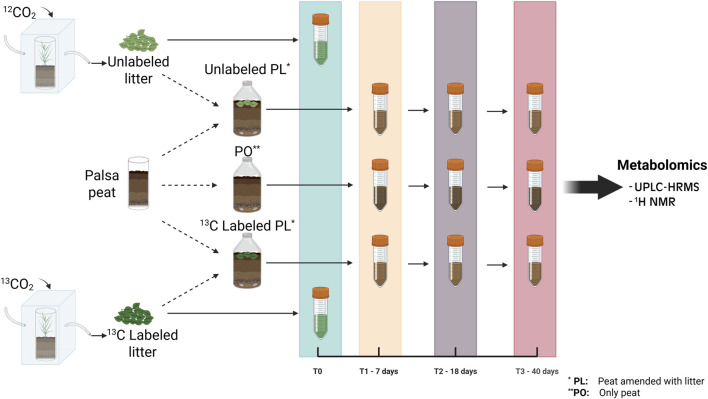
Schematic representation of the experimental design. Representative plants from the palsa were labeled *in vivo* by growing in chambers with air enriched in ^13^CO_2_. Labeled and unlabeled litter were then incubated with active layer palsa peat for up to 40 days. Peat-only (PO) samples and peat samples amended with labeled and unlabeled litter were collected after 7, 18, and 40 days for organic matter characterization, and metagenome sequencing whereas for Litter-only (LO) samples, we used the original T0 material to understand the starting organic matter composition. Figure created with BioRender.

An oxic incubation experiment was performed to identify potential litter degradation pathways and to track key intermediate and end products of litter decomposition. Active layer peat samples were collected from the top 5 cm of the same palsa sites as the plants used for the production of labeled litter. These samples were then homogenized with minimal aeration and refrigerated overnight before use. All incubation flasks were prepared by adding ∼38 mL of palsa active layer peat. The experimental treatments for the incubation included peat-only (PO) samples, and samples amended with either ^13^C labeled or unlabeled ground litter (peat and litter, PL). For the amended samples, we used the ground plant material described above, adding 0.58 g of this processed litter (dry weight) to reach a ratio of 0.2 g of litter/g of dry peat equivalent (based on bulk density measures of field moist peat), chosen to match litter deposition rates previously observed in the field (Hough et al., 2022). Two replicates from each treatment were prepared resulting in a total of 18 samples. Samples were incubated at field water content (81% w/v) with an aerobic headspace at 10 °C (normal temperature range for surface soil in the summer) except during gas sampling. Field water content was maintained by weighing samples at days 7 and 18 and adding enough water to return the samples to their initial weight at the beginning of the incubation (0.06–0.24 g depending on the mass lost of each sample). Two replicate samples per treatment were destructively harvested on each of days 7 (T1), 18 (T2) and 40 (T3) for organic matter (OM) characterization. Upon harvesting, these samples were immediately frozen and maintained at −20 °C prior to analysis. Litter-only samples (LO) (dry ground material that was used for the incubation) were also stored at −20 °C and analyzed alongside the treatment samples for downstream analyses. A schematic of the methods is presented in Figure 1. All sample names, codes and treatments are specified in Supplementary Table S1.

### 2.4 Gas isotopic measurements and calculations of gas production

Incubation jars were sampled on days 1, 3, 5, and thereafter every 5 days until day 40 for total and isotopic measurement of CO_2_. Gas measurements were performed using a Picarro G2131-i Cavity Ringdown Spectrometer equipped with a Small Sample Isotope Module (SSIM). In all cases, incubations were first injected with 40 mL of 400 ppm CO_2_ standard gas (with delta = −33.8), then direct injection to the SSIM was performed in dual injection mode (for a total of 40 mL gas sampled (Dickinson et al., 2017). Incubation jars were then flushed with the same standard gas for 5 min and resealed.

The amount of CO_2_ produced from the litter in amended samples (PL) was calculated as:

$$Litter\;respiration=\frac{F_{Net}*\left(R_{Net}-R_{Soil}\right)}{R_{Litter}-R_{Soil}}$$

Where $F_{Net}$ = total CO_2_ flux, $R_{Net}$ = ^13^CO_2_/total CO_2_ flux, $R_{Soil}$ = mean ^13^CO_2_/total CO_2_ flux of unamended peat incubations (PO samples), and $R_{Litter}$ = ^13^C/total C of amended labeled incubations (labeled PL samples). The amount of CO_2_ produced from peat in amended samples (PL) was calculated as the total CO_2_ flux minus litter respiration. The production of CO_2_ due to priming was calculated as the difference between the CO_2_ flux due to peat only (i.e., total CO_2_ flux minus CO_2_ flux due to litter) of amended peat incubations (PL samples) and the total CO_2_ flux of unamended peat incubations (PO samples).

### 2.5 Organic matter characterization

Frozen active layer peat samples (4 timepoints, 3 treatments, 2 replicates each (except T0), total = 19 samples) and litter samples (labeled and unlabeled, total = 2 samples) were shipped to the Environmental Molecular Sciences Laboratory (EMSL) at the Pacific Northwest National Laboratory (PNNL), Richland, WA, United States for processing. Metabolites were extracted using milliQ water following the protocol developed by (Tfaily et al., 2017). We specifically selected water extraction to target labile and moderately labile metabolites that would be most relevant to early-stage decomposition processes. This approach mimics natural field conditions where water infiltration from precipitation events facilitates the leaching and subsequent decomposition of soil organic matter compounds in peat systems. While water extraction primarily captures polar, water-soluble compounds and may not comprehensively represent the more recalcitrant organic matter fractions, it provides an environmentally relevant snapshot of the bioavailable metabolite pool that would be most actively involved in microbial degradation and priming responses. Briefly, 100 mg of peat from each sample was suspended in 1 mL of milliQ water in a 2 mL Microsolv glass vial and shaken for 2 h at room temperature. Samples were then spun down at 4430 RFC using a centrifuge and the supernatant was aspirated and collected in a new vial. Extracts were split for both ^1^H NMR and UPLC-HRMS analysis (discussed below).

Changes in metabolome molecular composition were characterized with UPLC-HRMS consisting of a Waters Acquity ultra-performance liquid chromatograph (UPLC) (Waters Corporation, Milford, MA, USA) connected to a 21 T Fourier transform Ion Cyclotron resonance (21T FT ICR) high resolution mass spectrometer (HRMS) (Bruker Daltonics, Billerica, MA, United States) (Shaw et al., 2016; Walker et al., 2017; Fudyma et al., 2019), located at EMSL, PNNL, Richland, WA, United States. The high mass resolution power of the 21T FT ICR HRMS was utilized in differentiating between the ^13^C labeled and unlabeled compounds with accurate mass that facilitated tracking the plant litter degradation process.

For the UPLC step, 10 µL of sample extracts were injected into a Zorbax C18 column (0.5 mm × 150 mm × 5 µm) (Agilent Technologies, Inc., Santa Clara, CA, United States) Samples were eluted using two solutions: 5 mM aqueous ammonium formate solution (solvent A) and a 5 mM ammonium formate in mass spectrometry grade methanol solution (solvent B). Elution was done at a flow rate of 0.2 mL/min with a 60 min linear gradient of solvent B from 5% to 95% followed by isocratic elution at 95% of solvent B for 10 min. The 21T FT ICR HRMS was equipped with a heated electrospray ionization source operated in negative ionization mode. The capillary voltage was −3500 V, and the precursor ions and fragmentation features were acquired with accurate mass using collisionally induced dissociation with a collision energy of 40 eV.

### 2.6 UPLC-HRMS spectra processing and annotation

Raw UPLC-HRMS spectra were processed with Compound Discoverer (version 3.3) (ThermoFisher Scientific, Waltham, MA, USA) using a modified version of the Stable Isotope Labeling preset workflow. This workflow enabled feature detection, molecular formula generation, isotopic pattern comparison, fragmentation pattern assignment and comparison, and annotation using online databases. Mass spectrometry data was filtered to include masses between 100 and 1,200 Da. Retention time alignment was performed using an adaptive curve model with a tolerance of 5 ppm. The minimum intensity threshold for feature detection was 2 × 10^6^ and a signal-to-noise ratio greater than 3.

**FIGURE 2:**
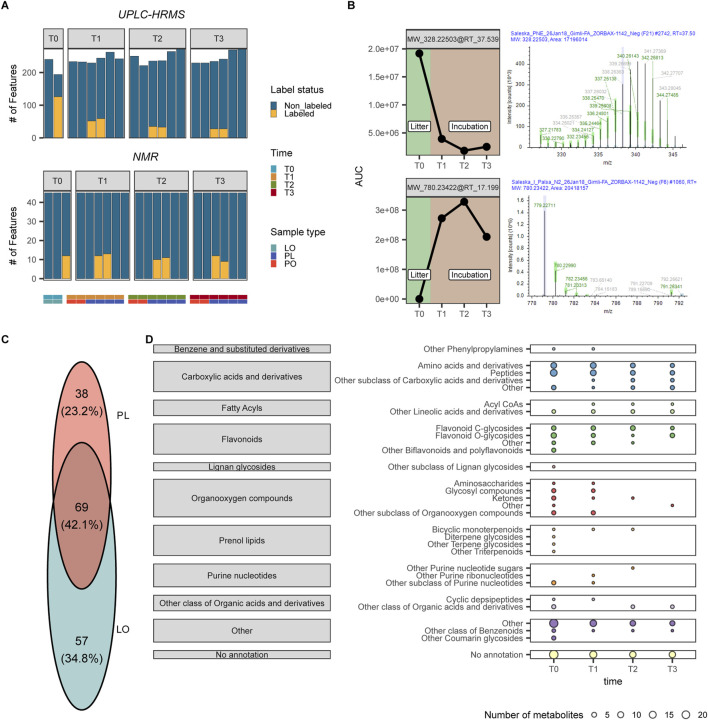
**(A)** Number of detected features using UPLC-HRMS and ^1^H NMR. The number of labeled metabolites that were detected per sample is shown in yellow. **(B)** Temporal dynamics and isotopologue occurrence pattern of features MW_328.22503@RT_37.539 (top) (more abundant in the litter) and MW_780.23422@RT_17.199 (bottom) (more abundant after incubation). Left: Changes in compound abundance across different time points, from initial litter (LO T0) through peat incubation periods (PL T1, T2, T3). Values represent mean abundance. Right: Isotopic distribution pattern of this compound in the ^13^C-labeled litter sample, showing mass shift patterns that indicate carbon incorporation from labeled source material. The multiple peaks represent different isotopologues with varying numbers of ^13^C atoms incorporated into the molecular structure. **(C)** Venn diagram showing how many metabolites were detected to be labeled only in the LO samples, the PL samples, and how many were labeled in both sample types. **(D)** Metabolic pathway dynamics across decomposition timeline. Bubble plot illustrating the number and distribution of ^13^C-labeled metabolites detected across different molecular classes and subclasses at each incubation time point (T0, T1, T2, T3). Bubble size corresponds to the count of unique labeled metabolites identified within each category, revealing temporal shifts in metabolic pathway activity during litter decomposition. This visualization highlights which biochemical pathways become activated or suppressed throughout the degradation process.

In the SIAM approach, ^13^C-labeled metabolites occur together with their natural counterparts and as such they can be detected in groups using UPLC-HRMS (Wei et al., 2017; Tian et al., 2018) because the labeled and unlabeled features share the same chromatographic retention time, have specific exact mass differences and diagnostic intensity ratios (Neumann et al., 2014) (see Figure 2B). Stable isotope integration with untargeted metabolomics serves two essential functions. It enhances metabolite identification accuracy and reveals biochemical pathway relationships (Creek et al., 2012). In our analytical workflow, we used Compound Discoverer software to first detect and determine elemental composition of metabolites using the unlabeled reference samples. The software then identified the isotopically labeled counterparts of these metabolites and calculated their fractional label incorporation after correcting for natural abundance. We exported the abundance data for both labeled and unlabeled metabolites in CSV format for subsequent analysis.

Annotation of the detected UPLC-HRMS metabolites to obtain level 2 (putatively annotated compound using public libraries without reference standards) and level 3 annotations (putatively characterized compound classes) (Sumner et al., 2007) was made using a combination of spectral library searches against the mzCloud database, and the GNPS database (Wang et al., 2016) (MS2 databases) (high confidence level 2 annotations). MS1-based database searches with ChemSpider (Pence and Williams, 2010) (low-confidence level 2 annotations), and *in silico* prediction of structures and molecular classes were made using the SIRIUS (Dührkop et al., 2019) and CANOPUS (Dührkop et al., 2021) fragmentation analysis pipelines (level 3 annotations).

For level 2 annotations, Compound Discoverer was used to perform spectral library searches against the mzCloud database, and MS1-based searches using ChemSpider (Pence and Williams, 2010) to annotate metabolites against the BioCyc (Karp et al., 2019), ChEBI (Degtyarenko et al., 2008), ChemBank (Seiler et al., 2008), Human Metabolome Database (Wishart et al., 2007; 2022), KEGG (Kanehisa et al., 2002) and MassBank (Horai et al., 2010) databases based on their mass and assigned molecular formula. Because of the high number of unannotated metabolites with MS2 available, their spectra were exported as Mascot Generic Format (MGF) files using a custom R script that combines the *MSnbase* package (Gatto et al., 2021) and the ThermoRawFileParser (Hulstaert et al., 2020). Exported MS2 spectra were used for annotation against the GNPS database using their own Library Search Tool (Wang et al., 2016). Annotations from the Kyoto Encyclopedia of Genes and Genomes (Kanehisa et al., 2002) were retrieved manually from annotated metabolites.

For metabolites that have a level 2 annotation, InChIKeys were retrieved from PubChem by querying its REST API web service PUG-REST (Kim et al., 2018). These metabolites were classified using ClassyFire (Djoumbou Feunang et al., 2016) based on their molecular structure (InChiKeys). For metabolites that did not have any annotation, but have MS2 available, molecular class was predicted using the CANOPUS (Dührkop et al., 2021) tool in the SIRIUS metabolomics framework (Dührkop et al., 2019).

### 2.7 ^1^H NMR

For ^1^H NMR analysis, water extract samples (180 µL) from the frozen peat samples and controls described in Section 2.5 (n = 21 samples) - were combined with 2,2-dimethyl-2-silapentane- 5-sulfonate-d6 (DSS-d6) in D2O (20 μL, 5 mM) and thoroughly mixed prior to transfer to 3 mm NMR tubes. One-dimensional (1D) ^1^H NMR spectra were acquired on a Varian 600 MHz VNMRS spectrometer equipped with a 5-mm triple-resonance (HCN) cold probe at a regulated temperature of 298 K (Varian, Inc., Palo Alto, CA, USA). The 90° ^1^H pulse was calibrated prior to the measurement of each sample. The one-dimensional (1D) ^1^H spectra were acquired using a nuclear Overhauser effect spectroscopy (NOESY) pulse sequence with a spectral width of 12 ppm and 512 transients. The NOESY mixing time was 100 ms, and the acquisition time was 4 s, followed by a relaxation delay of 1.5 s during which presaturation of the water signal was applied. Time-domain free induction decays (57,472 total points) were zero filled to 131,072 total points prior to Fourier transform. Chemical shifts were referenced to the ^1^H methyl signal in DSS-d_6_ at 0 ppm. The 1D ^1^H spectra were manually processed, assigned metabolite identification, and quantified using Chenomx NMR Suite 8.3. Candidate metabolites present in each of the complex mixtures were determined by library matching to Chenomx and custom in-house databases by matching the chemical shift, J-coupling, and intensity of experimental signals. Quantification was based on fitted metabolite signals relative to the internal standard. (DSS-d6). Signal to noise ratios (S/N) were measured using MestReNova 14 with the limit of quantification set to 10 and the limit of detection set to 3. In several cases further corroboration of metabolite identity was made using standard 2-D experiments such as ^1^H/^13^C - heteronuclear correlation (HSQC) experiments or 2-D ^1^H/^1^H Total Correlation spectroscopy (TOCSY).

### 2.8 Statistical analysis

Carbon isotopes (^13^C and ^12^C) share identical electronic configurations, meaning labeled molecules maintain the same chemical characteristics as their unlabeled counterparts. The isotope effects from ^13^C substitution are negligible and do not significantly alter biological systems or introduce measurement errors in metabolomics analysis (Freund and Hegeman, 2017). This principle allowed us to treat labeled and unlabeled LO and PL samples as analytical replicates for statistical purposes. All statistical analyses were performed in RStudio (version 2023.12) using R (version 4.3.2) (R Core Team, 2023) as detailed below, all visualizations were produced with ggplot2 (Wickham, 2016). The code for all the analysis done in this study can be found in its Github repository (https://github.com/Coayala/palsa_metabolomics).

To assess the impact of litter addition on the metabolome profile of active layer palsa peat, we analyzed the normalized abundance of detected features using both multivariate and univariate statistical approaches. For all statistical analysis, the abundances of the metabolites detected by UPLC-HRMS or ^1^H NMR were first normalized by median normalization and then pareto scaled. Differences between all samples were analyzed with a principal coordinate analysis (PCoA) based on “manhattan” distances using functions from the *vegan* R package (Oksanen et al., 2024). Additionally, the same distances were also used for hierarchical clustering analysis. To highlight the differences between PL samples and T1 and other samples from the incubation experiment, an additional non-metric multidimensional scaling (NMDS) (Kruskal, 1964) ordination was applied without considering LO samples. For both cases, a permutational analysis of variance (PERMANOVA) was applied to determine if sample type or time were the main drivers of the observed differences.

A Multiblock (s)PLS-DA (Singh et al., 2019) from the mixomics package (Rohart et al., 2017), was used to determine metabolites that can discriminate between PL and PO samples. This analysis uses an N-integration framework allowing for the simultaneous analysis of different multiomics datasets. To visualize the changes in abundance of discriminatory metabolites, the abundance of each metabolite was expressed as log2-fold change relative to its value in the PO samples at T0.

## 3 Results

### 3.1 Isotopic tracing identifies litter-derived nitrogen in soil amino acids and peptides

Analyzing the metabolome of litter (LO), peat (PO) and litter-amended peat (PL) samples at different time points (Supplementary Table S1) with the current UPLC-HRMS procedure, we detected a total of 320 features with masses ranging from 275 to 1,193 Da (Figure 2A) (Supplementary Table S2). Among these, 164 features (51.25%) showed incorporation of the ^13^C label from samples that received labeled litter (either labeled litter alone or peat with labeled litter), as evidenced by their mass differences and expected diagnostic ratios (Tian et al., 2018) (Supplementary Table S3).

We successfully annotated 103 features (32.19%) out of the 320 features with molecular structures. Since our analysis did not utilize an in-house library, all annotations should be considered level 2 confidence or lower. Of these annotations: 26 metabolites (8.21%) were assigned high-confidence annotations based on MS2 spectral matches against the mzCloud database, 9 metabolites (2.5%) were annotated using MS2 spectra matches from the GNPS database (Wang et al., 2016). The remaining 68 annotations (21.25%) were based on mass and molecular formula searches using ChemSpider.

For molecular classification, features with a putative chemical structure (103 features) were classified using ClassyFire (Djoumbou Feunang et al., 2016), with molecular classes assigned to 92 of them (28.75% of the total 320 features). Features lacking annotation but with available MS2 data were classified using CANOPUS (156 out of 320, 47.5%) (Dührkop et al., 2021). A total of 61 out of the total 320 features (19.06%) remained unnanotated and unclassified (Supplementary Table S4).

In addition to UPLC-HRMS, we utilized ^1^H NMR spectroscopy for structural elucidation of low-molecular-weight metabolites. This technique detected 45 metabolites with masses ranging from 32 to 355 Da, of which 18 (40%) showed isotopic labeling at one or more time points (Figure 2A) (Supplementary Tables S5, S6). The complementary use of UPLC-HRMS and ^1^H NMR enhanced our capacity to analyze the effects of litter addition, particularly given their differing optimal mass detection ranges. Analysis of labeled metabolites via UPLC-HRMS revealed diverse isotopologues with varying levels of ^13^C incorporation (Figure 2B). The LO samples exhibited the highest abundance of heavy isotopologues, consistent with enriched ^13^C levels in the palsa litter. The isotopologue profile fluctuated over time, with certain labeled metabolites restricted to litter samples and others appearing only at specific incubation time points (Figure 2C). The majority of detected labeled metabolites were categorized as amino acids and peptide-like compounds, with flavonoids representing the second most abundant group (Figure 2D). Nitrogen-rich compounds exhibited particularly distinct distribution patterns throughout the experiment. We observed that certain amino acids and peptides (both labeled and unlabeled variants) appeared exclusively in litter-only (LO) samples, while different nitrogen-containing compounds were uniquely present in peat-with-litter (PL) samples, indicating dynamic transformation processes of these biologically important molecules.

### 3.2 Rapid microbial response to litter inputs in palsa peat ecosystems

**FIGURE 3:**
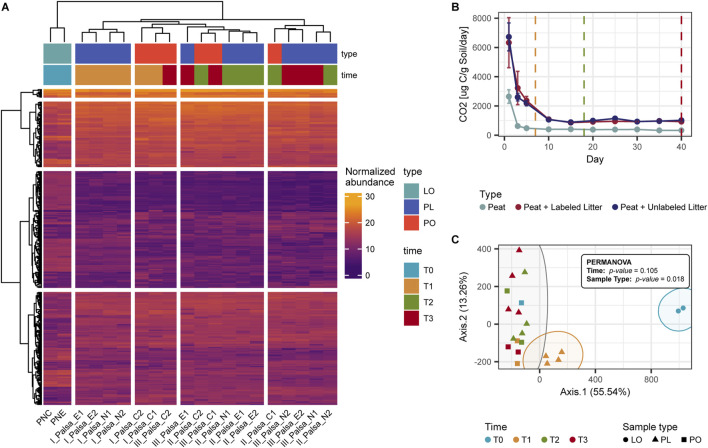
Effect of litter addition on the organic matter profile of the palsa peat. For this analysis, we treated both labeled and unlabeled samples as biological replicates to capture the broader metabolomic response **(A)** Heatmap and hierarchical clustering of the normalized abundances of the features detected with UPLC-HRMS **(B)** Mean CO_2_ production during the incubation (Hough, 2020). **(C)** PCoA ordination of the normalized UPLC-HRMS abundances showing how litter-amended samples at T1 cluster separately from unamended samples and litter-amended samples at other time points. LO: Litter-only samples, PL: peat samples amended with litter, PO: peat-only samples.

For the ordination analysis, we considered the entire set of metabolites, encompassing all detected features, and for litter-only (LO) and peat-amended with litter (PL) samples, we treated both labeled and unlabeled samples as replicates to capture the broader metabolomic response. Hierarchical clustering and ordination analysis revealed distinct differences in the metabolome profiles of the litter-only (LO) samples compared to the peat-only (PO) and litter-amended peat (PL) samples (Figures 3A,C). In particular, the organic matter profile of PL samples at T1 (7 days) differed significantly from all other PL and PO samples (Figures 3A,C). Permutational analysis of variance (PERMANOVA) confirmed that sample type, rather than incubation time, had a significant influence on metabolite abundance (p < 0.05). Notably, PL-T1 samples were significantly different from all others (p = 0.033; Supplementary Figure S1).

This distinct PL-T1 metabolomic signature corresponded with an early peak in CO_2_ production, which was higher in amended compared to unamended samples (consistent with Hough, 2020). Clustering analysis supported this, with PL-T1 samples forming a unique group, distinct from both unamended controls and other amended samples at later time points (Figure 3A). Over time, as microbial activity declined (Figure 3B), the metabolome of amended peat (PL) gradually resembled that of unamended controls (PO).

Litter addition also increased the relative abundance of several metabolite subclasses in PL samples, such as amines, amino acids, and purines (Supplementary Figure S2). These metabolites exhibited a rapid increase followed by a sharp decline in abundance after 14 days (Figures 3A,C), indicating transient accumulation and swift microbial utilization.

### 3.3 Transient old carbon mobilization following litter addition in palsa peat

**FIGURE 4:**
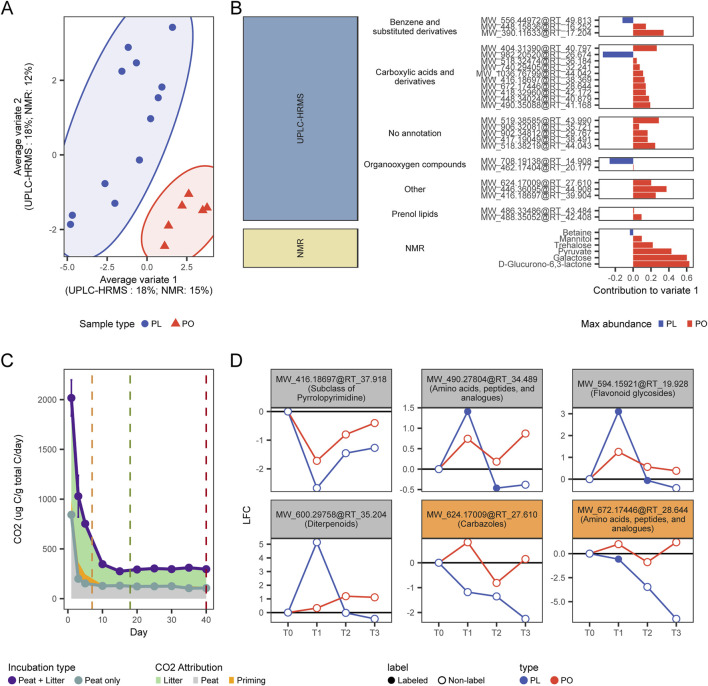
**(A)** Multiblock (s)PLS-DA ordination using both the UPLC-HRMS and ^1^H NMR datasets **(B)** Discriminatory features explaining the observed differences in the Variate 1 of the multiblock (s)PLS-DA ordination. **(C)** Origin of the measured CO_2_ fluxes. **(D)** Changes in abundance over time (expressed as log2 fold-changes (LFC) relative to T0) of metabolites potentially involved with priming. Metabolites highlighted in orange were determined as significant by the multiblock (s)PLS-DA.

To further elucidate the mechanisms of organic matter degradation triggered by the addition of litter, and using both labeled and unlabeled samples as biological replicates to enhance the robustness of our investigation, and confirm whether the observed patterns of gas flux measurements and changes in metabolomics profiles (Figure 3C) correspond to a transient priming effect, we employed a Multiblock sparse partial least squares discriminant analysis (Multiblock sPLS-DA) (Rohart et al., 2017), to contrast the metabolomics signatures of the amended (PL) and unamended peat samples (PO). This approach allowed us to integrate our UPLC-HRMS and ^1^H NMR datasets and identify discriminant variables whose abundance significantly differs between amended and unamended samples. This analysis allowed us to identify 20 discriminant UPLC-HRMS features and 6 discriminant ^1^H NMR features that accounted for 18% and 15% of the variation in metabolome profiles between PO and PL samples, respectively (Figure 4A) (Supplementary Table S7).

Most of these discriminatory features were more abundant in unamended samples compared to amended samples (Figure 4B). This suggests that the observed differences in the metabolome caused by litter addition is due to an enhancement of the degradation of existing peat metabolites or due to an inhibition of their natural accumulation, both of which can result in peat metabolites becoming less abundant in the PO samples. The potential breakdown of older peat metabolites caused by the effect of newly added nutrient sources such as litter inputs, may indicate that litter addition to the palsa peat is inducing a positive priming effect (Wild et al., 2014). Thus, these discriminatory features could serve as potential indicators of priming activity, which seems to corroborate the claims of positive priming occurrence that were based only on gas flux data (Figure 4C) (Hough, 2020).

Our results suggest that litter addition either enhanced degradation or suppressed accumulation of specific peat-derived compounds. For example, two unlabeled metabolites showed particularly notable responses: MW_624.17009@RT_27.610 and MW_672.17446@RT_28.644. Both consistently declined in PL samples throughout the incubation, eventually falling below their initial T0 levels. Their unlabeled status confirms they originated from native peat rather than added litter. The first compound was annotated as a carbazole (a recalcitrant polycyclic aromatic structure), while the second was identified as an N-acyl amino acid derivative. These classes of metabolites can be accumulated in the peat due to their complex structure or antimicrobial properties (Koshlaf and Ball, 2017; Lee et al., 2019), but can still be degraded by diverse bacterial groups across various ecological niches (Salam et al., 2017). The degradation of these peat metabolites may indicate their role as a reservoir of carbon or nitrogen that peat microbial communities can access under the correct conditions, such as an increased energy. In addition, at least four other non-discriminant metabolites, only assigned a molecular class level, were also found to decrease in abundance from T0 to T3 in PL samples (Figure 4B), despite an initial accumulation due to litter addition (evidenced by the presence of the ^13^C label). The reduction of all these metabolites, after the addition of amendments, corroborates a weak priming activity, where microbial communities used energy from litter decomposition (Wang and Roulet, 2017) to process otherwise recalcitrant peat-derived compounds (Figure 4D). However, as the labile fraction gets consumed, as shown by the overall decrease of amino acids and peptide derivatives (Supplementary Figure S3), microbial communities do not seem able to sustain the positive priming effect past the initial incubation stages (Figure 4C).

### 3.4 Diverse microbial strategies for litter metabolite utilization

To elucidate potential litter decomposition pathways, metabolites with molecular structure detected via UPLC-HRMS (103 metabolites) and ^1^H NMR (45 metabolites) across all samples regardless of labeling status, were mapped onto the KEGG database (Kanehisa et al., 2002). We identified 10 UPLC-HRMS metabolites and all ^1^H NMR metabolites (45 metabolites) involved in microbial pathways according to KEGG present in either LO or PL samples. Annotations from the KEGG database revealed that the UPLC-HRMS metabolites included flavonoids such as quercitrin, rutin, epicatechin, and myricitrin, which are small phenolic compounds produced by vegetation as secondary metabolites. These compounds are found typically in peatlands as plant secretions or litter inputs (Panis and Rompel, 2022).

**FIGURE 5:**
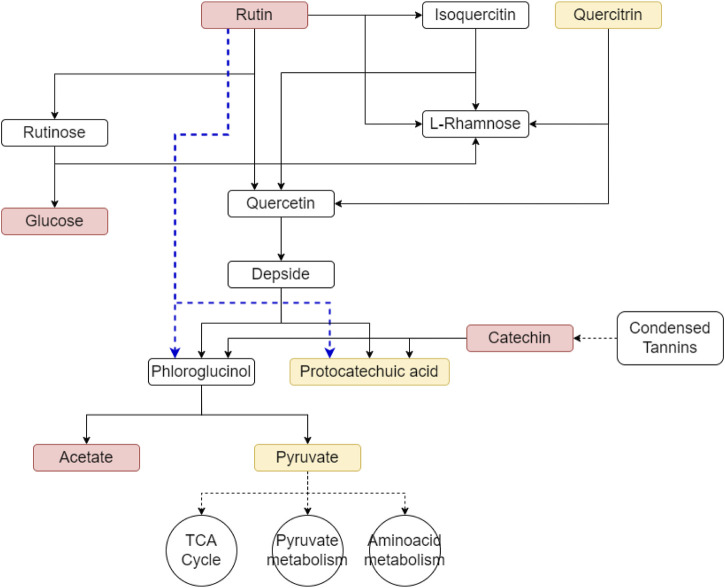
Potential degradation pathways for litter-derived flavonoids derived from the litter such as rutin can be degraded through the rutin catabolic pathway. Metabolites in red indicate those that were found labeled in our dataset, metabolites in yellow were present in the dataset but without label. The blue line indicates an alternate rutin degradation pathway that does not involve the formation of the intermediate quercetin.

Even though, tannins and other polyphenols can be difficult to degrade due to the formation of complexes or due to having antimicrobial properties (Lewis and Starkey, 1968), analysis of the labeled data allowed us to identify changes in their relative abundance, as well as the presence of labeled forms of these metabolites (e.g., rutin, epicatechin, and vitexin-2-o-beta-L-rhamnoside) at different time points throughout the incubation (Supplementary Figure S4). The observed changes in abundance of these metabolites confirms the ability of peatland microbial communities to decompose phenolic compounds (McGivern et al., 2021), and suggest that they are potentially transforming these metabolites through reactions associated with flavone and flavonol biosynthesis, as well as flavonoid degradation metabolic pathways (Figure 5).

Rutin, a common plant-derived flavonoid and one of the few well-characterized labeled metabolites detected in both LO and PL samples, was observed to decrease in abundance throughout the incubation period suggesting active decomposition by peat microbial communities. Glucose and protocatechuic acid (as protocatechuate), which are known degradation products of rutin, were also detected across all time points, even though only glucose showed evidence of labeling (Supplementary Figure S4). However, other expected intermediates of rutin degradation such as isoquercetin, quercetin, or phloroglucinol were not detected. Other key components of condensed tannins: (−) epicatechin and its dimer procyanidin B5 (Arunachalam et al., 2003) were also detected during our analysis across all time points. They initially showed a decrease in their abundance relative to litter inputs, however their concentrations increased by T2 and T3 (Supplementary Figure S4), potentially indicating dynamic degradation and accumulation processes.

^1^H NMR data also revealed the presence of several labeled and unlabeled metabolites, including sugars, amino acids, and small organic acids involved in central carbon metabolism (Supplementary Table S5). For instance, we observed an increase in pyruvate abundance coupled with a decrease in glucose levels, suggesting active glycolysis (Supplementary Figure S4). The presence of labeled glucose across all time points (Supplementary Table S6) suggested that it may have originated either from labeled litter inputs, or result from the degradation of labeled litter metabolites such as polyphenols or polysaccharides.

Several amino acids showed decreasing trends in abundance, while larger labeled peptides were uniquely identified in the UPLC-HRMS dataset for PL samples (Supplementary Figure S5), suggesting microbial synthesis from smaller litter-derived metabolites. Additionally, the use of ^13^C-labeled litter enabled the detection of plant-derived low molecular weight metabolites, including fructose, glucose, and sucrose, up to 40 days into the incubation. Unlabeled metabolites not originally detected in litter, such as mannitol and lactate, were also observed, as well as fermentation products and degradation intermediates like gallate and protocatechuate.

## 4 Discussion

The consistent detection of labeled features across our metabolomic datasets demonstrates that our ^13^C stable-isotope labeling protocol successfully enabled us to track litter-derived compounds through various transformation pathways during decomposition. Variability in ^13^C incorporation is expected, given influences such as metabolic flux and plant growth rate (Freund and Hegeman, 2017), and is reflected in the dynamic isotopologue profiles over time. The prominence of labeled metabolites in LO (litter only) samples supports initial enrichment in litter material (Wang and Jones, 2014), while the fluctuating presence of labeled compounds in the PL (litter-amended peat) suggests ongoing degradation and transformation processes, likely driven by microbial activity (Gilbert et al., 2022) and specific biological pathway preferences (Rasmussen and Hoffman, 2020).

A large portion of metabolites detected across all samples were annotated as amino acids and peptide derivatives, which aligns with expectations for RP-UPLC-HRMS in negative ion mode. The prevalence of these nitrogen-rich metabolites can also be attributed to their abundance in *Eriophorum spp*., a dominant component of palsa vegetation (Wilson et al., 2022). These compounds constitute a significant portion of the labile dissolved organic matter (DOM) pool (Marschner and Kalbitz, 2003), require minimal enzymatic breakdown, and thus are readily bioavailable (Lynch, 1982). They can also accumulate in the soil as products from microbial metabolism (Miltner et al., 2009).

The absence of certain amino acids and peptides in later time points of the incubation in unamended peat samples (PL) (Supplementary Figure S5) suggests their rapid decomposition (Sachse et al., 2005), consistent with their high turnover rates (Kielland et al., 2007), and roles as key sources of organic nitrogen in soils (Schulten and Schnitzer, 1997). Their disappearance (Supplementary Figure S5) likely reflects microbial utilization for energy and biosynthesis (Philben et al., 2015). In contrast, the presence of labeled amino acids and peptides exclusively in PL samples is indicative of microbial transformation processes, such as the biosynthesis of new metabolites from litter-derived precursors. Previous studies have indicated microbial proteases as key agents in degrading plant proteinaceous material (Wanek et al., 2010), releasing amino acids that can then be accumulated as microbial biomass (Schimel and Bennett, 2004; Kelleher et al., 2006; Jones and Kielland, 2012). These findings support the detection of PL-specific labeled peptides and underscore the importance of proteolytic activity in microbial decomposition of litter-derived organic matter. Together, these results demonstrate the utility of stable isotope-assisted metabolomics for tracing the origin and fate of nitrogen-containing metabolites in complex soil systems.

The rapid microbial response to litter addition in palsa peat was observed as a distinct metabolomic signature in PL-T1. This response aligns not only with CO_2_ release, but also with findings by Hough (2020), where 73% of the microbial community incorporated the ^13^C label by day 7, increasing to 82% by day 18. These “fast responders” dominated during early litter decomposition, which likely explains the strong deviation in metabolome profiles observed during the initial stages of incubation. The transient accumulation and subsequent disappearance of labile compounds such as amino acids, purines, and amines further underscored this rapid response. These metabolites, likely representing water-soluble plant derivatives, are rapidly metabolized by soil microbes, reflecting a dynamic cycle of nitrogen and carbon processing within the palsa active layer, consistent with previous reports of fast metabolic shifts following litter addition (Bourget et al., 2023).

As litter-derived labile compounds were depleted, microbial activity diminished, and the metabolome of amended samples began to resemble unamended controls. This transition mirrors the classic two-phase model of litter decomposition, where easily accessible metabolites drive early microbial activity (Fanin et al., 2014), followed by a slowdown as more recalcitrant materials like lignin and polysaccharides become dominant (Berg and Ekbohm, 1991; Kosheleva and Trofimov, 2008). The energy required to degrade lignin often comes from more labile carbon sources (Berg et al., 1984), explaining the observed pattern of initial activity followed by stabilization.

The return of the PL metabolome to a PO-like state also reflects peatland ecology, where the accumulation of partially decomposed organic matter is a defining feature (Clymo, 1996; Finlayson and Milton, 2018). Previous microbial community analysis conducted by (Hough, 2020) showed that while the dominant taxa (≥0.5% abundance) remained relatively stable, certain functional groups responded to litter addition. Notably, there was an increase in taxa associated with denitrification, such as Rhodanobacteraceae, and Burkholderiaceae (Hao et al., 2021; Takatsu et al., 2022), as well as those involved with carbohydrate degradation such as Sphingobacteriaceae (Pankratov et al., 2007). These findings complement our current metabolomic observations, highlighting how litter addition temporarily stimulates microbial metabolism and reshapes the chemical landscape of peat soils, even if these shifts are short-lived.

The Multiblock sPLS-DA analysis provided compelling evidence that litter addition induced temporary changes in the metabolome of palsa peat, with several peat-derived metabolites declining in abundance in amended samples. This pattern aligns with the concept of a priming effect, where the addition of labile substrates stimulates the breakdown of older, native organic matter (Wild et al., 2014). Importantly, our data corroborates the weak and transient priming effects that were inferred by the gas flux through the use of stable isotope-assisted metabolomics. We believe the discriminatory features revealed with this analysis could serve as potential indicators of priming activity, capable of signaling even small occurrences of this phenomenon and providing an additional line of evidence to previous observations relying only on gas data (Figure 4C).

This observation, along with our earlier findings on increased microbial activity and rapid shifts in the palsa peat metabolome, suggests microbial metabolic activation as the main priming mechanism following litter addition (Kuzyakov et al., 2000). It further supports the efficient processing of plant inputs in the palsa’s active layer and emphasizes the complex interactions between fresh litter and legacy organic matter. The potential priming effect indicated by these discriminatory features may contribute to the dynamic nature of carbon cycling in thawing permafrost ecosystems. The observed depletion of metabolites like the carbazole and N-acyl amino acid derivative (Figure 4D), indicates activation of degradation pathways for complex peat compounds. While such compounds are typically resistant to degradation (Koshlaf and Ball, 2017), or may serve antimicrobial roles (Lee et al., 2019), various bacteria can utilize them as carbon and nitrogen sources (Inoue et al., 2005; Nojiri and Omori, 2007; Salam et al., 2017). While their degradation likely contributes to increased carbon gas losses, the return to baseline microbial activity (Figure 4C) suggests that priming was minimal and short-lived. Similar transient positive priming effects have also been observed in other peatlands (Mastný et al., 2021), and aquatic systems (Steen et al., 2016; Textor et al., 2018).

The limited priming response observed in our study aligns with recent research suggesting low sensitivity of peat and organic layers to priming by plant-derived metabolites under oxic conditions (Wild et al., 2023). This reduced sensitivity is attributed to the inherently high soil organic carbon (SOC) content, high SOC/N ratios, and nitrogen limited nature of peat (Wild et al., 2023). In some ecosystems, plant-derived carbon can push microbial communities toward nitrogen limitation, prompting extracellular enzyme production (Craine et al., 2007). In our system, while plant-derived organic nitrogen can provoke CO2 release (Wild et al., 2023), the shift in C:N ratios has little to no effect on increasing native organic matter decomposition, as microbial communities of the palsa are already adapted to nitrogen-limiting conditions (Song et al., 2022). Given that *E. vaginatum* litter has high C:N and low protein content (Hough et al., 2022; Wilson et al., 2022), its addition may have only transiently stimulated microbial activity (Figures 3B, 4C), reinforcing the idea that microbial activation, rather than nutrient mining, drives the brief priming effect observed in these permafrost peatlands.

Previous research has shown that under oxic conditions, microbes tend to prioritize decomposing more energetically favorable and accessible substrates like fresh litter over native peat organic matter, which requires more energy investment to break down (Fontaine et al., 2003). This preferential utilization could explain why, after the initial stimulation of microbial activity and CO_2_ release, the priming effect was not sustained once the labile litter fraction was consumed. For example, many metabolites classified as amino acid derivatives or peptides, which can be considered highly nutritious, seem to have an overall decreasing trend throughout the incubation (Supplementary Figure S3). Nonetheless, the absence of a stronger priming signal may also be attributed to the relatively short duration of our incubation experiment. A study summarizing global patterns of the priming effect reported a mean experiment duration of 128 days across 802 compiled datasets (Mo et al., 2022). A longer-term experiment tracking CO_2_ production, as well as the microbial communities structure and activity, could potentially reveal a delayed priming effect, manifesting after microbes deplete the fresh litter and switch to decomposing older peat organic matter, possibly through late activation of microbial nitrogen mining (Perveen et al., 2019).

Overall, the combination of preferential substrate utilization by specific microbial enzyme systems under oxic conditions, along with the high SOC and nitrogen limited conditions of the peat, likely explains the limited priming of native peat decomposition observed in our incubation experiment. Furthermore, our use of stable isotope assisted metabolomics allowed us to identify priming signals that could not be confirmed by the use of gas flux data alone (Hough, 2020), providing a more complete understanding of the effect of litter addition upon peat-stored metabolites. These findings underscore the complex interplay between fresh litter inputs, existing peat organic matter, and microbial communities in subarctic peatland ecosystems, highlighting how the use of emerging analytical tools can enable more detailed studies needed to fully elucidate the dynamics of carbon cycling in these sensitive environments.

Besides providing additional evidence regarding the occurrence of the priming effect, the analysis of ^13^C-labeled metabolomics data also revealed potential litter utilization pathways in the palsa peatlands. The decrease in abundance of flavonoids such as rutin, epicatechin, and myricitrin in PL samples (Supplementary Figure S4) suggests active microbial metabolism of these plant secondary metabolites. While the enzyme latch theory suggests oxygen limitations in the peatlands might inhibit their degradation (Freeman et al., 2001), recent studies have shown that wetland microbial communities can decompose a diverse array of phenolic metabolites (McGivern et al., 2021).

For example, rutin, a defense compound exuded by plants, can be degraded in the soil by fungal (*Aspergillus* and *Penicillium*) (Westlake et al., 1959; Mamma et al., 2004), and bacterial (*Streptomyces*) (Merkens et al., 2008; Yang et al., 2009) taxa. The typical rutin catabolic pathway involves enzymatic reactions catalyzed by glycosidase, dioxygenase, and esterase, resulting in the production of sugars (rhamnose and glucose), carbon monoxide, and phenolic compounds like phloroglucinol and protocatechuic acid (Tranchimand et al., 2010). Interestingly, while we detected both glucose (in labeled form) and protocatechuic acid (as protocatechuate), we did not observe other intermediate degradation products such as isoquercetin, quercetin, or phloroglucinol. This may suggest an alternative rutin degradation pathway in the palsa peat that involves the direct cleavage of the heterocyclic ring (Westlake et al., 1959), bypassing quercetin formation and releasing phloroglucinol and protocatechuic acid (Westlake et al., 1959; Yang et al., 2009). The absence of detectable phloroglucinol in our incubations may be explained by its rapid aerobic degradation into acetate and pyruvate (Armstrong and Patel, 1994). Alternatively, the lack of detection of other intermediate products could be due to their rapid consumption or the limitations of the analytical approach used.

Similar to rutin, other tannin components such as (−)epicatechin and its dimer procyanidin B5 can also be decomposed by heterocyclic C-ring fission, resulting in the production of phloroglucinol and phenylacetate or benzoic acid derivatives (McGivern et al., 2021). Interestingly, although these metabolites declined initially, their abundance rose at later incubation stages (Supplementary Figure S4), likely due to ongoing tannin cleavage (Boominathan and Mahadevan, 1985; Mutabaruka et al., 2007) throughout the incubation. As polyphenol degradation feeds into central metabolism (Figure 5), these pathways are likely important for microbial nutrient acquisition from litter in the palsa peat (McGivern et al., 2021).

The increase in pyruvate and decrease in glucose observed in the amended samples (PL) during the incubation (Supplementary Figure S4) further suggests microbial engagement in glycolysis. Labeled glucose likely originated from polysaccharides or polyphenols in the litter, degraded by extracellular enzymes like hemicellulases and cellulases (Burns et al., 2013). The pyruvate generated during glycolysis can be utilized in fermentation processes to produce acetate or in the synthesis of amino acids such as alanine, valine, and leucine. The use of these sugars in central metabolism for energy production and amino acid synthesis aligns with the concurrent decrease in free amino acids and emergence peptides in UPLC-HRMS data (Supplementary Figures S4, S5).

Moreover, the persistent availability of labeled sugars (e.g., glucose, fructose, sucrose) up to 40 days post-litter addition may have influenced the lack of sustained priming effects observed (as discussed in Section 3.3). This availability supports microbial preference for labile substrates and aligns with studies showing that decomposition of leaves from vascular plants such as *E. vaginatum* can release substantial amounts of labile metabolites, including organic acids, sugars, and amino acids (Mastný et al., 2018). Our analysis also revealed the presence of unlabeled metabolites not detected in the original litter, including fermentation products such as mannitol and lactate. These compounds, which can be produced during carbohydrate degradation (Wisselink et al., 2002) may represent additional degradation products from partially labeled and unlabeled litter metabolites. Similarly, the accumulation of gallate and protocatechuate appears to result from the degradation of plant derived phenolic compounds (Fan et al., 2017; Arafat et al., 2020).

## 5 Conclusion

In this study, we employed an emerging approach combining stable isotope labeling of *E. vaginatum* litter with advanced metabolomics techniques (SIAM) to elucidate the metabolic pathways involved in organic matter decomposition within palsa peat. This innovative methodology has provided new insights into the fate of litter derived compounds and their impact on peatland carbon cycling. Our research revealed that litter inputs significantly contribute to the organic nitrogen pool in palsa peat, primarily through amino acids and peptide derivatives. These bioavailable compounds serve as readily accessible nutrient sources for microbial communities. We successfully identified flavonoids and other polyphenols originating from the litter, either directly synthesized by plants or produced through tannin hydrolysis. While evidence of polyphenol degradation was observed, likely through heterocyclic C-ring fission, their accumulation of some of them in amended peat samples suggests a role in limiting overall litter decomposition.

Our results corroborated previous findings showing that under oxic conditions (Hough, 2020), palsa peat microbial communities rapidly utilize the more bioavailable compounds present in *E. vaginatum* litter (Wilson et al., 2022). This was evidenced by the dynamic changes in the organic matter profile during the first 7 days, followed by a gradual return to a profile resembling unamended peat. Importantly, we demonstrated that priming effects in this system are transient and minimal, with microbial communities showing a brief period of enhanced activity followed by a rapid return to baseline metabolism. This transient nature suggests that while litter inputs temporarily stimulate microbial activity, they do not sustain long-term acceleration of stored carbon decomposition. By combining stable isotope labeling with comparative metabolome profiling of amended and unamended samples, we identified potential metabolites undergoing weak positive priming. This molecular level evidence confirmed the existence of priming processes that were not detectable through CO_2_ gas measurements alone. The novelty of our approach lies in its ability to provide a comprehensive, molecular-level understanding of litter decomposition processes in peatland ecosystems. We identified potential key metabolites (e.g., litter-derived polyphenols) and pathways (e.g., degradation of flavonoids via heterocyclic C-ring fission) governing litter degradation. This level of detail goes beyond previous studies that relied on chemical composition and energetic analysis to understand litter bioavailability influences on carbon release from thawing permafrost systems.

As climate change may facilitate the displacement of *Sphagnum* spp. mosses by vascular plants, the faster-decomposing litter from these plants is likely to increase CO_2_ fluxes, potentially creating a positive climate feedback loop. Our findings underscore the importance of litter quality and composition in influencing SOM degradation and accumulation across different sub-habitats of the permafrost gradient. Furthermore, our research highlights how technological advancements in analytical tools can enable a more detailed view into the complex interactions between litter, microbes, and peat organic matter. The use of these cutting-edge technologies provide means for tracking labeled metabolites across degradation processes, improving our understanding of carbon cycling and the emissions of climate active gases, uncovering processes that could not be fully confirmed with traditional environmental measurements. Future studies building on this approach will be crucial to unravel the complex interactions between plant communities, microbial metabolism, and greenhouse gas production in these sensitive ecosystems.

## Data Availability

The metabolite abundance and annotation data generated for the study are included in the article Supplementary Materials. Raw mass spectrometry data is included in the OSF repository: https://osf.io/5r3z6/. The scripts used to analyze the data and generate the figures for this paper can be found in its GitHub repository (https://github.com/Coayala/palsa_metabolomics).

## References

[B1] AminiTabrizi R. Wilson R. M. Fudyma J. D. Hodgkins S. B. Heyman H. M. Rich V. I. (2020). Controls on soil organic matter degradation and subsequent greenhouse gas emissions across a permafrost thaw gradient in northern Sweden. Front. Earth Sci. Chin. 8, 557961. 10.3389/feart.2020.557961

[B2] AminiTabrizi R. Graf-Grachet N. Chu R. K. Toyoda J. G. Hoyt D. W. Hamdan R. (2023). Microbial sensitivity to temperature and sulfate deposition modulates greenhouse gas emissions from peat soils. Glob. Chang. Biol. 29, 1951–1970. 10.1111/gcb.16614 36740729

[B3] Antala M. Juszczak R. van der Tol C. Rastogi A. (2022). Impact of climate change-induced alterations in peatland vegetation phenology and composition on carbon balance. Sci. Total Environ. 827, 154294. 10.1016/j.scitotenv.2022.154294 35247401

[B4] Arafat Y. Ud Din I. Tayyab M. Jiang Y. Chen T. Cai Z. (2020). Soil sickness in aged tea plantation is associated with a shift in microbial communities as a result of plant polyphenol accumulation in the tea gardens. Front. Plant Sci. 11, 601. 10.3389/fpls.2020.00601 32547573 PMC7270330

[B5] Armstrong S. M. Patel T. R. (1994). Microbial degradation of phloroglucinol and other polyphenolic compounds. J. Basic Microbiol. 34, 123–135. 10.1002/jobm.3620340208 8014845

[B6] Arunachalam M. Raj M. M. Mohan N. Mahadevan A. (2003). Biodegradation of catechin. Proceedings-Indian Natl. Sci. Acad. Part B 69, 353–370. Available online at: https://citeseerx.ist.psu.edu/document?repid=rep1&type=pdf&doi=d99cc5067255baca6ba2e4775d6494e8fbe41c4f.

[B7] Bell M. C. Ritson J. P. Verhoef A. Brazier R. E. Templeton M. R. Graham N. J. D. (2018). Sensitivity of peatland litter decomposition to changes in temperature and rainfall. Geoderma 331, 29–37. 10.1016/j.geoderma.2018.06.002

[B8] Berg B. Ekbohm G. (1991). Litter mass-loss rates and decomposition patterns in some needle and leaf litter types. Long-term decomposition in a Scots pine forest. VII. VII. *Can. J. Bot.* 69, 1449–1456. 10.1139/b91-187

[B9] Berg B. Ekbohm G. McClaugherty C. (1984). Lignin and holocellulose relations during long-term decomposition of some forest litters. Long-term decomposition in a Scots pine forest. IV. Can. J. Bot. 62, 2540–2550. 10.1139/b84-345

[B10] Bhatnagar J. M. Peay K. G. Treseder K. K. (2018). Litter chemistry influences decomposition through activity of specific microbial functional guilds. Ecol. Monogr. 88, 429–444. 10.1002/ecm.1303

[B11] Bingeman C. W. Varner J. E. Martin W. P. (1953). The effect of the addition of organic materials on the decomposition of an organic soil. Soil Sci. Soc. Am. J. 17, 34–38. 10.2136/sssaj1953.03615995001700010008x

[B12] Boominathan K. Mahadevan A. (1985). “Evidence for the existence of catabolic plasmid in Pseudomonas solanacearum. Conference of the association of Proceedings. 85.

[B13] Bourget M. Y. Fanin N. Fromin N. Hättenschwiler S. Roumet C. Shihan A. (2023). Plant litter chemistry drives long‐lasting changes in the catabolic capacities of soil microbial communities. Funct. Ecol. 37, 2014–2028. 10.1111/1365-2435.14379

[B14] Burns R. G. DeForest J. L. Marxsen J. Sinsabaugh R. L. Stromberger M. E. Wallenstein M. D. (2013). Soil enzymes in a changing environment: current knowledge and future directions. Soil Biol. biochem. 58, 216–234. 10.1016/j.soilbio.2012.11.009

[B15] Buttler A. Robroek B. J. M. Laggoun‐Défarge F. Jassey V. E. J. Pochelon C. Bernard G. (2015). Experimental warming interacts with soil moisture to discriminate plant responses in an ombrotrophic peatland. J. Veg. Sci. 26, 964–974. 10.1111/jvs.12296

[B16] Chen K. Xiang Y. Yan X. Li Z. Qin R. Sun J. (2022). Global tracking of transformation products of environmental contaminants by 2H-labeled stable isotope-assisted metabolomics. Anal. Chem. 94, 7255–7263. 10.1021/acs.analchem.2c00500 35510918

[B17] Chiapusio G. Jassey V. E. J. Bellvert F. Comte G. Weston L. A. Delarue F. (2018). Sphagnum species modulate their phenolic profiles and Mycorrhizal colonization of surrounding Andromeda polifolia along Peatland microhabitats. J. Chem. Ecol. 44, 1146–1157. 10.1007/s10886-018-1023-4 30294748

[B18] Clymo R. S. (1996). Assessing the accumulation of carbon in peatlands. *Northern peatlands in global climate change* . Available online at: https://www.osti.gov/etdeweb/servlets/purl/458161#page=208.

[B19] Cory A. B. Wilson R. M. Holmes M. E. Riley W. J. Li Y.-F. Tfaily M. M. (2025). A climatically significant abiotic mechanism driving carbon loss and nitrogen limitation in peat bogs. Sci. Rep. 15, 2560. 10.1038/s41598-025-85928-w 39833269 PMC11747108

[B20] Craine J. M. Morrow C. Fierer N. (2007). Microbial nitrogen limitation increases decomposition. Ecology 88, 2105–2113. 10.1890/06-1847.1 17824441

[B21] Creek D. J. Chokkathukalam A. Jankevics A. Burgess K. E. V. Breitling R. Barrett M. P. (2012). Stable isotope-assisted metabolomics for network-wide metabolic pathway elucidation. Anal. Chem. 84, 8442–8447. 10.1021/ac3018795 22946681 PMC3472505

[B22] Dargie G. C. Lewis S. L. Lawson I. T. Mitchard E. T. A. Page S. E. Bocko Y. E. (2017). Age, extent and carbon storage of the central Congo Basin peatland complex. Nature 542, 86–90. 10.1038/nature21048 28077869

[B23] Degtyarenko K. de Matos P. Ennis M. Hastings J. Zbinden M. McNaught A. (2008). ChEBI: a database and ontology for chemical entities of biological interest. Nucleic Acids Res. 36, D344–D350. 10.1093/nar/gkm791 17932057 PMC2238832

[B24] Dickinson D. Bodé S. Boeckx P. (2017). System for *δ* ^13^C–CO_2_ and *x*CO_2_ analysis of discrete gas samples by cavity ring-down spectroscopy. Atmos. Meas. Tech. 10, 4507–4519. 10.5194/amt-10-4507-2017

[B25] Dieleman C. M. Branfireun B. A. McLaughlin J. W. Lindo Z. (2015). Climate change drives a shift in peatland ecosystem plant community: implications for ecosystem function and stability. Glob. Chang. Biol. 21, 388–395. 10.1111/gcb.12643 24957384

[B26] Djoumbou Feunang Y. Eisner R. Knox C. Chepelev L. Hastings J. Owen G. (2016). ClassyFire: automated chemical classification with a comprehensive, computable taxonomy. J. Cheminform. 8, 61. 10.1186/s13321-016-0174-y 27867422 PMC5096306

[B27] Dührkop K. Fleischauer M. Ludwig M. Aksenov A. A. Melnik A. V. Meusel M. (2019). SIRIUS 4: a rapid tool for turning tandem mass spectra into metabolite structure information. Nat. Methods 16, 299–302. 10.1038/s41592-019-0344-8 30886413

[B28] Dührkop K. Nothias L.-F. Fleischauer M. Reher R. Ludwig M. Hoffmann M. A. (2021). Systematic classification of unknown metabolites using high-resolution fragmentation mass spectra. Nat. Biotechnol. 39, 462–471. 10.1038/s41587-020-0740-8 33230292

[B29] Fan D.-M. Fan K. Yu C.-P. Lu Y.-T. Wang X.-C. (2017). Tea polyphenols dominate the short-term tea (Camellia sinensis) leaf litter decomposition. J. Zhejiang Univ. Sci. B 18, 99–108. 10.1631/jzus.B1600044 28124839 PMC5296227

[B30] Fanin N. Hättenschwiler S. Fromin N. (2014). Litter fingerprint on microbial biomass, activity, and community structure in the underlying soil. Plant Soil 379, 79–91. 10.1007/s11104-014-2051-7

[B31] Fiehn O. (2002). “Metabolomics — the link between genotypes and phenotypes,” in Functional genomics. Editor Town C. (Dordrecht: Springer Netherlands), 155–171. 10.1007/978-94-010-0448-0_11 11860207

[B32] Finlayson C. Milton G. R. (2018). “Peatlands,” in The wetland book II: distribution, description and conservation (Springer), 227–244. Available online at: https://researchoutput.csu.edu.au/en/publications/peatlands.

[B33] Fofana A. Anderson D. McCalley C. K. Hodgkins S. Wilson R. M. Cronin D. (2022). Mapping substrate use across a permafrost thaw gradient. Soil Biol. biochem. 175, 108809. 10.1016/j.soilbio.2022.108809

[B34] Fontaine S. Mariotti A. Abbadie L. (2003). The priming effect of organic matter: a question of microbial competition? Soil Biol. biochem. 35, 837–843. 10.1016/S0038-0717(03)00123-8

[B35] Freeman C. Ostle N. Kang H. (2001). An enzymic “latch” on a global carbon store. Nature 409, 149. 10.1038/35051650 11196627

[B36] Freund D. M. Hegeman A. D. (2017). Recent advances in stable isotope-enabled mass spectrometry-based plant metabolomics. Curr. Opin. Biotechnol. 43, 41–48. 10.1016/j.copbio.2016.08.002 27610928

[B37] Fudyma J. D. Lyon J. AminiTabrizi R. Gieschen H. Chu R. K. Hoyt D. W. (2019). Untargeted metabolomic profiling of Sphagnum fallax reveals novel antimicrobial metabolites. Plant Direct 3, e00179. 10.1002/pld3.179 31742243 PMC6848953

[B38] Fudyma J. D. Toyoda J. G. Chu R. K. Weitz K. K. Heyman H. M. Eder E. (2021). Sequential abiotic‐biotic processes drive organic carbon transformation in peat bogs. J. Geophys. Res. Biogeosci. 126, e2020JG006079. 10.1029/2020jg006079

[B39] Gatto L. Gibb S. Rainer J. (2021). MSnbase, efficient and elegant R-Based processing and visualization of raw mass spectrometry data. J. Proteome Res. 20, 1063–1069. 10.1021/acs.jproteome.0c00313 32902283

[B40] Gilbert A. Nakagawa M. Taguchi K. Zhang N. Nishida A. Yoshida N. (2022). Hydrocarbon cycling in the Tokamachi Mud Volcano (Japan): insights from isotopologue and metataxonomic analyses. Microorganisms 10, 1417. 10.3390/microorganisms10071417 35889138 PMC9323770

[B41] Gorham E. (1991). Northern peatlands: role in the carbon cycle and probable responses to climatic warming. Ecol. Appl. 1, 182–195. 10.2307/1941811 27755660

[B42] Guenet B. Leloup J. Raynaud X. Bardoux G. Abbadie L. (2010). Negative priming effect on mineralization in a soil free of vegetation for 80 years. Eur. J. Soil Sci. 61, 384–391. 10.1111/j.1365-2389.2010.01234.x

[B43] Hao D.-C. Wang L. Gao W. Xie H. Bao X. Jia Z. (2021). Disentangling effects of Moisture/gas regimes on microbial community, network configuration and nitrogen turnover of Black soil. Eurasian Soil Sci. 54, S42–S61. 10.1134/S1064229322030073

[B44] Hättenschwiler S. Vitousek P. M. (2000). The role of polyphenols in terrestrial ecosystem nutrient cycling. Trends Ecol. Evol. 15, 238–243. 10.1016/s0169-5347(00)01861-9 10802549

[B45] Hodgkins S. B. Tfaily M. M. McCalley C. K. Logan T. A. Crill P. M. Saleska S. R. (2014). Changes in peat chemistry associated with permafrost thaw increase greenhouse gas production. Proc. Natl. Acad. Sci. U. S. A. 111, 5819–5824. 10.1073/pnas.1314641111 24711402 PMC4000816

[B46] Holmes M. E. Crill P. M. Burnett W. C. McCalley C. K. Wilson R. M. Frolking S. (2022). Carbon accumulation, flux, and fate in stordalen mire, a permafrost peatland in transition. Glob. Biogeochem. Cycles 36, e2021GB007113. 10.1029/2021gb007113

[B47] Horai H. Arita M. Kanaya S. Nihei Y. Ikeda T. Suwa K. (2010). MassBank: a public repository for sharing mass spectral data for life sciences. J. Mass Spectrom. 45, 703–714. 10.1002/jms.1777 20623627

[B48] Hou R. Gan L. Guan F. Wang Y. Li J. Zhou S. (2021). Bioelectrochemically enhanced degradation of bisphenol S: mechanistic insights from stable isotope-assisted investigations. iScience 24, 102014. 10.1016/j.isci.2020.102014 33490921 PMC7809511

[B49] Hough M. (2020). Tracing the new carbon cycle from plant inputs to microbial outputs across an arctic permafrost thaw gradient. The University of Arizona. dissertation. Available online at: https://search.proquest.com/openview/00465c7a1db1500fe5e2f0d36ac1c7f7/1?pq-origsite=gscholar&cbl=18750&diss=y.

[B50] Hough M. McCabe S. Vining S. R. Pickering Pedersen E. Wilson R. M. Lawrence R. (2022). Coupling plant litter quantity to a novel metric for litter quality explains C storage changes in a thawing permafrost peatland. Glob. Chang. Biol. 28, 950–968. 10.1111/gcb.15970 34727401 PMC9298822

[B51] Hulstaert N. Shofstahl J. Sachsenberg T. Walzer M. Barsnes H. Martens L. (2020). ThermoRawFileParser: modular, scalable, and cross-platform RAW file conversion. J. Proteome Res. 19, 537–542. 10.1021/acs.jproteome.9b00328 31755270 PMC7116465

[B52] Inoue K. Habe H. Yamane H. Omori T. Nojiri H. (2005). Diversity of carbazole-degrading bacteria having the car gene cluster: isolation of a novel gram-positive carbazole-degrading bacterium. FEMS Microbiol. Lett. 245, 145–153. 10.1016/j.femsle.2005.03.009 15796992

[B53] Johansson T. Malmer N. Crill P. M. Friborg T. Åkerman J. H. Mastepanov M. (2006). Decadal vegetation changes in a northern peatland, greenhouse gas fluxes and net radiative forcing: decadal changes of carbon flux and forcing. Glob. Chang. Biol. 12, 2352–2369. 10.1111/j.1365-2486.2006.01267.x

[B54] Jones D. L. Kielland K. (2012). Amino acid, peptide and protein mineralization dynamics in a taiga forest soil. Soil Biol. biochem. 55, 60–69. 10.1016/j.soilbio.2012.06.005

[B55] Kanehisa M. Goto S. Kawashima S. Nakaya A. (2002). The KEGG databases at GenomeNet. Nucleic. Acids Res. 30, 42–46. 10.1093/nar/30.1.42 11752249 PMC99091

[B56] Karp P. D. Billington R. Caspi R. Fulcher C. A. Latendresse M. Kothari A. (2019). The BioCyc collection of microbial genomes and metabolic pathways. Brief. Bioinform. 20, 1085–1093. 10.1093/bib/bbx085 29447345 PMC6781571

[B57] Kaštovská E. Straková P. Edwards K. Urbanová Z. Bárta J. Mastný J. (2018). Cotton-Grass and blueberry have opposite effect on peat characteristics and nutrient transformation in Peatland. Ecosystems 21, 443–458. 10.1007/s10021-017-0159-3

[B58] Kelleher B. P. Simpson M. J. Simpson A. J. (2006). Assessing the fate and transformation of plant residues in the terrestrial environment using HR-MAS NMR spectroscopy. Geochim. Cosmochim. Acta 70, 4080–4094. 10.1016/j.gca.2006.06.012

[B59] Kellogg J. Kang S. (2020). Metabolomics, an essential tool in exploring and harnessing microbial chemical ecology. Phytobiomes J. 4, 195–210. 10.1094/PBIOMES-04-20-0032-RVW

[B60] Kielland K. McFarland J. W. Ruess R. W. Olson K. (2007). Rapid cycling of organic nitrogen in taiga Forest ecosystems. Ecosystems 10, 360–368. 10.1007/s10021-007-9037-8

[B61] Kim S. Thiessen P. A. Cheng T. Yu B. Bolton E. E. (2018). An update on PUG-REST: RESTful interface for programmatic access to PubChem. Nucleic Acids Res. 46, W563-W570–W570. 10.1093/nar/gky294 29718389 PMC6030920

[B62] Kosheleva Y. P. Trofimov S. Y. (2008). Characteristics of the biochemical composition of plant litter at different stages of decomposition (according to thermal analysis data). Biol. Bull. Russ. Acad. Sci. 35, 64–69. 10.1134/s106235900801010x 18491564

[B63] Koshlaf E. Ball A. S. (2017). Soil bioremediation approaches for petroleum hydrocarbon polluted environments. AIMS Microbiol. 3, 25–49. 10.3934/microbiol.2017.1.25 31294147 PMC6604977

[B64] Kruskal J. B. (1964). Nonmetric multidimensional scaling: a numerical method. Psychometrika 29, 115–129. 10.1007/bf02289694

[B65] Kuzyakov Y. Friedel J. K. Stahr K. (2000). Review of mechanisms and quantification of priming effects. Soil Biol. biochem. 32, 1485–1498. 10.1016/S0038-0717(00)00084-5

[B66] Lal R. (2008). Carbon sequestration. Philos. Trans. R. Soc. Lond. B Biol. Sci. 363, 815–830. 10.1098/rstb.2007.2185 17761468 PMC2610111

[B67] Lal R. (2010). Managing Soils and ecosystems for mitigating anthropogenic carbon emissions and advancing global food security. Bioscience 60, 708–721. 10.1525/bio.2010.60.9.8

[B68] Lee C.-M. Kim S.-Y. Yoon S.-H. Kim J.-B. Yeo Y.-S. Sim J.-S. (2019). Characterization of a novel antibacterial N-acyl amino acid synthase from soil metagenome. J. Biotechnol. 294, 19–25. 10.1016/j.jbiotec.2019.01.017 30771442

[B69] Lewis J. A. Starkey R. L. (1968). Vegetable tannins, their decomposition and effects on decomposition of some organic compounds. Soil Sci. 106, 241–247. 10.1097/00010694-196810000-00001

[B70] Liang J. Zhou Z. Huo C. Shi Z. Cole J. R. Huang L. (2018). More replenishment than priming loss of soil organic carbon with additional carbon input. Nat. Commun. 9, 3175. 10.1038/s41467-018-05667-7 30093611 PMC6085371

[B71] Liu X. Locasale J. W. (2017). Metabolomics: a primer. Trends biochem. Sci. 42, 274–284. 10.1016/j.tibs.2017.01.004 28196646 PMC5376220

[B72] Liu X.-J. A. Sun J. Mau R. L. Finley B. K. Compson Z. G. van Gestel N. (2017). Labile carbon input determines the direction and magnitude of the priming effect. Appl. Soil Ecol. 109, 7–13. 10.1016/j.apsoil.2016.10.002

[B73] Liu X.-J. A. Finley B. K. Mau R. L. Schwartz E. Dijkstra P. Bowker M. A. (2020). The soil priming effect: consistent across ecosystems, elusive mechanisms. Soil Biol. biochem. 140, 107617. 10.1016/j.soilbio.2019.107617

[B74] Lunt P. H. Fyfe R. M. Tappin A. D. (2019). Role of recent climate change on carbon sequestration in peatland systems. Sci. Total Environ. 667, 348–358. 10.1016/j.scitotenv.2019.02.239 30833238

[B75] Lynch J. M. (1982). Limits to microbial growth in soil. Microbiology 128, 405–410. 10.1099/00221287-128-2-405

[B76] Malmer N. Johansson T. Olsrud M. Christensen T. R. (2005). Vegetation, climatic changes and net carbon sequestration in a North-Scandinavian subarctic mire over 30 years. Glob. Chang. Biol. 0, 1895–1909. 10.1111/j.1365-2486.2005.01042.x

[B77] Mamma D. Kalogeris E. Hatzinikolaou D. G. Lekanidou A. Kekos D. Macris B. J. (2004). Biochemical characterization of the multi-enzyme System produced by Penicillium decumbens grown on rutin. Food Biotechnol. 18, 1–18. 10.1081/FBT-120030382

[B78] Marschner B. Kalbitz K. (2003). Controls of bioavailability and biodegradability of dissolved organic matter in soils. Geoderma 113, 211–235. 10.1016/S0016-7061(02)00362-2

[B79] Mastný J. Kaštovská E. Bárta J. Chroňáková A. Borovec J. Šantrůčková H. (2018). Quality of DOC produced during litter decomposition of peatland plant dominants. Soil Biol. biochem. 121, 221–230. 10.1016/j.soilbio.2018.03.018

[B80] Mastný J. Bárta J. Kaštovská E. Picek T. (2021). Decomposition of peatland DOC affected by root exudates is driven by specific r and K strategic bacterial taxa. Sci. Rep. 11, 18677. 10.1038/s41598-021-97698-2 34548501 PMC8455546

[B81] McCalley C. K. Woodcroft B. J. Hodgkins S. B. Wehr R. A. Kim E.-H. Mondav R. (2014). Methane dynamics regulated by microbial community response to permafrost thaw. Nature 514, 478–481. 10.1038/nature13798 25341787

[B82] McGivern B. B. Tfaily M. M. Borton M. A. Kosina S. M. Daly R. A. Nicora C. D. (2021). Decrypting bacterial polyphenol metabolism in an anoxic wetland soil. Nat. Commun. 12, 2466. 10.1038/s41467-021-22765-1 33927199 PMC8084988

[B83] Merkens H. Kappl R. Jakob R. P. Schmid F. X. Fetzner S. (2008). Quercetinase QueD of Streptomyces sp. FLA, a monocupin dioxygenase with a preference for nickel and cobalt. Biochemistry 47, 12185–12196. 10.1021/bi801398x 18950192

[B84] Miltner A. Kindler R. Knicker H. Richnow H.-H. Kästner M. (2009). Fate of microbial biomass-derived amino acids in soil and their contribution to soil organic matter. Org. Geochem. 40, 978–985. 10.1016/j.orggeochem.2009.06.008

[B85] Mitsch W. J. Bernal B. Nahlik A. M. Mander Ü. Zhang L. Anderson C. J. (2013). Wetlands, carbon, and climate change. Landsc. Ecol. 28, 583–597. 10.1007/s10980-012-9758-8

[B86] Mo F. Ren C. Yu K. Zhou Z. Phillips R. P. Luo Z. (2022). Global pattern of soil priming effect intensity and its environmental drivers. Ecology 103, e3790. 10.1002/ecy.3790 35718753

[B87] Mondav R. McCalley C. K. Hodgkins S. B. Frolking S. Saleska S. R. Rich V. I. (2017). Microbial network, phylogenetic diversity and community membership in the active layer across a permafrost thaw gradient. Environ. Microbiol. 19, 3201–3218. 10.1111/1462-2920.13809 28574203

[B88] Moore T. Basiliko N. (2006). Decomposition in boreal peatlands. *Boreal peatland ecosystems* . Available online at: https://link.springer.com/content/pdf/10.1007/978-3-540-31913-9.pdf#page=144.

[B89] Mutabaruka R. Hairiah K. Cadisch G. (2007). Microbial degradation of hydrolysable and condensed tannin polyphenol–protein complexes in soils from different land-use histories. Soil Biol. biochem. 39, 1479–1492. 10.1016/j.soilbio.2006.12.036

[B90] Neumann N. K. N. Lehner S. M. Kluger B. Bueschl C. Sedelmaier K. Lemmens M. (2014). Automated LC-HRMS(/MS) approach for the annotation of fragment ions derived from stable isotope labeling-assisted untargeted metabolomics. Anal. Chem. 86, 7320–7327. 10.1021/ac501358z 24965664 PMC4126838

[B91] Nichols J. E. Peteet D. M. (2019). Rapid expansion of northern peatlands and doubled estimate of carbon storage. Nat. Geosci. 12, 917–921. 10.1038/s41561-019-0454-z

[B92] Nojiri H. Omori T. (2007). Carbazole metabolism by pseudomonads. 107–145. 10.1007/978-1-4020-6097-7_5

[B93] Norby R. J. Childs J. Hanson P. J. Warren J. M. (2019). Rapid loss of an ecosystem engineer: sphagnum decline in an experimentally warmed bog. Ecol. Evol. 9, 12571–12585. 10.1002/ece3.5722 31788198 PMC6875578

[B94] Oksanen J. Simpson G. L. Blanchet F. G. Kindt R. Legendre P. Minchin P. R. (2024). Vegan: community ecology package. Available online at: https://github.com/vegandevs/vegan.

[B95] Olefeldt D. Roulet N. T. (2012). Effects of permafrost and hydrology on the composition and transport of dissolved organic carbon in a subarctic peatland complex. J. Geophys. Res. 117. 10.1029/2011JG001819

[B96] Painter T. J. (1991). Lindow man, tollund man and other peat-bog bodies: the preservative and antimicrobial action of Sphagnan, a reactive glycuronoglycan with tanning and sequestering properties. Carbohydr. Polym. 15, 123–142. 10.1016/0144-8617(91)90028-B

[B97] Panis F. Rompel A. (2022). The novel role of tyrosinase enzymes in the storage of globally significant amounts of carbon in wetland ecosystems. Environ. Sci. Technol. 56, 11952–11968. 10.1021/acs.est.2c03770 35944157 PMC9454253

[B98] Pankratov T. A. Tindall B. J. Liesack W. Dedysh S. N. (2007). Mucilaginibacter paludis gen. nov., sp. Nov. and Mucilaginibacter gracilis sp. Nov., pectin-xylan- and laminarin-degrading members of the family Sphingobacteriaceae from acidic Sphagnum peat bog. Int. J. Syst. Evol. Microbiol. 57, 2349–2354. 10.1099/ijs.0.65100-0 17911309

[B99] Pence H. E. Williams A. (2010). ChemSpider: an online chemical information resource. J. Chem. Educ. 87, 1123–1124. 10.1021/ed100697w

[B100] Perveen N. Barot S. Maire V. Cotrufo M. F. Shahzad T. Blagodatskaya E. (2019). Universality of priming effect: an analysis using thirty five soils with contrasted properties sampled from five continents. Soil Biol. biochem. 134, 162–171. 10.1016/j.soilbio.2019.03.027

[B101] Philben M. Holmquist J. MacDonald G. Duan D. Kaiser K. Benner R. (2015). Temperature, oxygen, and vegetation controls on decomposition in a James Bay peatland. Glob. Biogeochem. Cycles 29, 729–743. 10.1002/2014gb004989

[B102] Qian Y. Chen Z. Wang J. Peng M. Zhang S. Yan X. (2023). H/D exchange coupled with 2H-labeled stable isotope-assisted metabolomics discover transformation products of contaminants of emerging concern. Anal. Chem. 95, 12541–12549. 10.1021/acs.analchem.3c02833 37574906

[B103] Qiu C. Zhu D. Ciais P. Guenet B. Peng S. (2020). The role of northern peatlands in the global carbon cycle for the 21st century. Glob. Ecol. Biogeogr. 29, 956–973. 10.1111/geb.13081

[B104] R Core Team (2023). R: a Language and environment for statistical computing. Available online at: https://www.R-project.org/.

[B105] Rantanen M. Karpechko A. Y. Lipponen A. Nordling K. Hyvärinen O. Ruosteenoja K. (2022). The Arctic has warmed nearly four times faster than the globe since 1979. Commun. Earth Environ. 3, 168–10. 10.1038/s43247-022-00498-3

[B106] Rasmussen C. Hoffman D. W. (2020). Intramolecular distribution of 13C/12C isotopes in amino acids of diverse origins. Amino Acids 52, 955–964. 10.1007/s00726-020-02863-y 32594254

[B107] Ristok C. Leppert K. N. Franke K. Scherer-Lorenzen M. Niklaus P. A. Wessjohann L. A. (2017). Leaf litter diversity positively affects the decomposition of plant polyphenols. Plant Soil 419, 305–317. 10.1007/s11104-017-3340-8

[B108] Rohart F. Gautier B. Singh A. Lê Cao K.-A. (2017). mixOmics: an R package for ’omics feature selection and multiple data integration. PLoS Comput. Biol. 13, e1005752. 10.1371/journal.pcbi.1005752 29099853 PMC5687754

[B109] Sachse A. Henrion R. Gelbrecht J. Steinberg C. E. W. (2005). Classification of dissolved organic carbon (DOC) in river systems: influence of catchment characteristics and autochthonous processes. Org. Geochem. 36, 923–935. 10.1016/j.orggeochem.2004.12.008

[B110] Salam L. B. Ilori M. O. Amund O. O. (2017). Properties, environmental fate and biodegradation of carbazole. 3 Biotech. 7, 111. 10.1007/s13205-017-0743-4 28567624 PMC5451359

[B111] Schimel J. P. Bennett J. (2004). Nitrogen mineralization: challenges of a changing paradigm. Ecology 85, 591–602. 10.1890/03-8002

[B112] Schulten H.-R. Schnitzer M. (1997). The chemistry of soil organic nitrogen: a review. Biol. Fertil. Soils 26, 1–15. 10.1007/s003740050335

[B113] Seiler K. P. George G. A. Happ M. P. Bodycombe N. E. Carrinski H. A. Norton S. (2008). ChemBank: a small-molecule screening and cheminformatics resource database. Nucleic Acids Res. 36, D351–D359. 10.1093/nar/gkm843 17947324 PMC2238881

[B114] Shaw J. B. Lin T.-Y. Leach F. E. Tolmachev A. V. Tolić N. Robinson E. W. (2016). 21 tesla fourier Transform ion Cyclotron resonance mass spectrometer greatly expands mass spectrometry toolbox. J. Am. Soc. Mass Spectrom. 27, 1929–1936. 10.1007/s13361-016-1507-9 27734325

[B115] Singh A. Shannon C. P. Gautier B. Rohart F. Vacher M. Tebbutt S. J. (2019). DIABLO: an integrative approach for identifying key molecular drivers from multi-omics assays. Bioinformatics 35, 3055–3062. 10.1093/bioinformatics/bty1054 30657866 PMC6735831

[B116] Smith D. M. Screen J. A. Deser C. Cohen J. Fyfe J. C. García-Serrano J. (2019). The Polar Amplification Model Intercomparison Project (PAMIP) contribution to CMIP6: investigating the causes and consequences of polar amplification. Geosci. Model Dev. 12, 1139–1164. 10.5194/gmd-12-1139-2019

[B117] Song Y. Cheng X. Song C. Li M. Gao S. Liu Z. (2022). Soil CO2 and N2O emissions and microbial abundances altered by temperature rise and nitrogen addition in active-layer soils of permafrost peatland. Front. Microbiol. 13, 1093487. 10.3389/fmicb.2022.1093487 36583043 PMC9792967

[B118] Stalheim T. Ballance S. Christensen B. E. Granum P. E. (2009). Sphagnan--a pectin-like polymer isolated from Sphagnum moss can inhibit the growth of some typical food spoilage and food poisoning bacteria by lowering the pH. J. Appl. Microbiol. 106, 967–976. 10.1111/j.1365-2672.2008.04057.x 19187129

[B119] Steen A. D. Quigley L. N. M. Buchan A. (2016). Evidence for the priming effect in a planktonic estuarine microbial community. Front. Mar. Sci. 3, 177438. 10.3389/fmars.2016.00006

[B120] Sumner L. W. Amberg A. Barrett D. Beale M. H. Beger R. Daykin C. A. (2007). Proposed minimum reporting standards for chemical analysis Chemical Analysis Working Group (CAWG) Metabolomics Standards Initiative (MSI). Metabolomics 3, 211–221. 10.1007/s11306-007-0082-2 24039616 PMC3772505

[B121] Takatsu Y. Miyamoto T. Tahvanainen T. Hashidoko Y. (2022). Nitrous oxide emission in response to pH from degrading Palsa mire peat due to permafrost thawing. Curr. Microbiol. 79, 56. 10.1007/s00284-021-02690-8 34982223

[B122] Textor S. R. Guillemette F. Zito P. A. Spencer R. G. M. (2018). An assessment of dissolved organic carbon biodegradability and priming in blackwater systems. J. Geophys. Res. Biogeosci. 123, 2998–3015. 10.1029/2018jg004470

[B123] Tfaily M. M. Chu R. K. Toyoda J. Tolić N. Robinson E. W. Paša-Tolić L. (2017). Sequential extraction protocol for organic matter from soils and sediments using high resolution mass spectrometry. Anal. Chim. Acta 972, 54–61. 10.1016/j.aca.2017.03.031 28495096

[B124] Tfaily M. M. Wilson R. M. Brewer H. M. Chu R. K. Heyman H. M. Hoyt D. W. (2019). Single-throughput complementary high-resolution analytical techniques for characterizing complex natural organic matter mixtures. J. Vis. Exp. 10.3791/59035 30663714

[B125] Tian Z. Vila J. Yu M. Bodnar W. Aitken M. D. (2018). Tracing the biotransformation of polycyclic aromatic hydrocarbons in contaminated soil using stable isotope-assisted metabolomics. Environ. Sci. Technol. Lett. 5, 103–109. 10.1021/acs.estlett.7b00554 31572742 PMC6767928

[B126] Tranchimand S. Brouant P. Iacazio G. (2010). The rutin catabolic pathway with special emphasis on quercetinase. Biodegradation 21, 833–859. 10.1007/s10532-010-9359-7 20419500

[B127] van Breemen N. (1995). How Sphagnum bogs down other plants. Trends Ecol. Evol. 10, 270–275. 10.1016/0169-5347(95)90007-1 21237035

[B128] Walker L. R. Tfaily M. M. Shaw J. B. Hess N. J. Paša-Tolić L. Koppenaal D. W. (2017). Unambiguous identification and discovery of bacterial siderophores by direct injection 21 Tesla Fourier transform ion cyclotron resonance mass spectrometry. Metallomics 9, 82–92. 10.1039/c6mt00201c 27905613

[B129] Wanek W. Mooshammer M. Blöchl A. Hanreich A. Richter A. (2010). Determination of gross rates of amino acid production and immobilization in decomposing leaf litter by a novel 15N isotope pool dilution technique. Soil Biol. biochem. 42, 1293–1302. 10.1016/j.soilbio.2010.04.001

[B130] Wang Z. Jones A. D. (2014). Profiling of stable isotope enrichment in specialized metabolites using liquid chromatography and multiplexed nonselective collision-induced dissociation. Anal. Chem. 86, 10600–10607. 10.1021/ac502205y 25300033

[B131] Wang Z. Roulet N. (2017). Comparison of plant litter and peat decomposition changes with permafrost thaw in a subarctic peatland. Plant Soil 417, 197–216. 10.1007/s11104-017-3252-7

[B132] Wang M. Carver J. J. Phelan V. V. Sanchez L. M. Garg N. Peng Y. (2016). Sharing and community curation of mass spectrometry data with Global Natural Products Social Molecular Networking. Nat. Biotechnol. 34, 828–837. 10.1038/nbt.3597 27504778 PMC5321674

[B133] Ward S. E. Orwin K. H. Ostle N. J. Briones J. I. Thomson B. C. Griffiths R. I. (2015). Vegetation exerts a greater control on litter decomposition than climate warming in peatlands. Ecology 96, 113–123. 10.1890/14-0292.1 26236896

[B134] Wardle D. A. Yeates G. W. Barker G. M. Bonner K. I. (2006). The influence of plant litter diversity on decomposer abundance and diversity. Soil Biol. biochem. 38, 1052–1062. 10.1016/j.soilbio.2005.09.003

[B135] Wei X. Lorkiewicz P. K. Shi B. Salabei J. K. Hill B. G. Kim S. (2017). Analysis of stable isotope assisted metabolomics data acquired by high resolution mass spectrometry. Anal. Methods 9, 2275–2283. 10.1039/C7AY00291B 28674558 PMC5492990

[B136] Westlake D. W. Talbot G. Blakley E. R. Simpson F. J. (1959). Microbiol decomposition of rutin. Can. J. Microbiol. 5, 621–629. 10.1139/m59-076 13844170

[B137] Wickham H. (2016). ggplot2: elegant graphics for data analysis. Available online at: https://ggplot2.tidyverse.org.

[B138] Wild B. Schnecker J. Alves R. J. E. Barsukov P. Bárta J. Capek P. (2014). Input of easily available organic C and N stimulates microbial decomposition of soil organic matter in arctic permafrost soil. Soil Biol. biochem. 75, 143–151. 10.1016/j.soilbio.2014.04.014 25089062 PMC4064687

[B139] Wild B. Li J. Pihlblad J. Bengtson P. Rütting T. (2019). Decoupling of priming and microbial N mining during a short-term soil incubation. Soil Biol. biochem. 129, 71–79. 10.1016/j.soilbio.2018.11.014

[B140] Wild B. Monteux S. Wendler B. Hugelius G. Keuper F. (2023). Circum-Arctic peat soils resist priming by plant-derived compounds. Soil Biol. biochem. 180, 109012. 10.1016/j.soilbio.2023.109012

[B141] Wilhelm R. Szeitz A. Klassen T. L. Mohn W. W. (2014). Sensitive, efficient quantitation of 13C-enriched nucleic acids *via* ultrahigh-performance liquid chromatography-tandem mass spectrometry for applications in stable isotope probing. Appl. Environ. Microbiol. 80, 7206–7211. 10.1128/AEM.02223-14 25217022 PMC4249172

[B142] Wilhelm R. C. Barnett S. E. Swenson T. L. Youngblut N. D. Koechli C. N. Bowen B. P. (2022). Tracing carbon metabolism with stable isotope metabolomics reveals the legacy of diverse carbon sources in soil. Appl. Environ. Microbiol. 88, e0083922. 10.1128/aem.00839-22 36300927 PMC9680644

[B143] Wilson R. M. Tfaily M. M. Kolton M. Johnston E. R. Petro C. Zalman C. A. (2021). Soil metabolome response to whole-ecosystem warming at the spruce and Peatland Responses under changing Environments experiment. Proc. Natl. Acad. Sci. U. S. A. 118, e2004192118. 10.1073/pnas.2004192118 34161254 PMC8237682

[B144] Wilson R. M. Hough M. A. Verbeke B. A. Hodgkins S. B. IsoGenie C. Chanton J. P. (2022). Plant organic matter inputs exert a strong control on soil organic matter decomposition in a thawing permafrost peatland. Sci. Total Environ. 820, 152757. 10.1016/j.scitotenv.2021.152757 35031367

[B145] Wishart D. S. Tzur D. Knox C. Eisner R. Guo A. C. Young N. (2007). HMDB: the human metabolome database. Nucleic Acids Res. 35, D521–D526. 10.1093/nar/gkl923 17202168 PMC1899095

[B146] Wishart D. S. Guo A. Oler E. Wang F. Anjum A. Peters H. (2022). HMDB 5.0: the Human Metabolome Database for 2022. Nucleic Acids Res. 50, D622–D631. 10.1093/nar/gkab1062 34986597 PMC8728138

[B147] Wisselink H. W. Weusthuis R. A. Eggink G. Hugenholtz J. Grobben G. J. (2002). Mannitol production by lactic acid bacteria: a review. Int. Dairy J. 12, 151–161. 10.1016/S0958-6946(01)00153-4

[B148] Yang C.-H. Huang Y.-C. Chen C.-Y. (2009). Degradation of rutin by Thermoactinomyces vulgaris and other thermophilic compost isolates. J. Agric. Food Chem. 57, 5095–5099. 10.1021/jf900617z 19489631

