# Supplementary material for: Tracing priming effects in palsa peat carbon dynamics using a stable isotope-assisted metabolomics approach
Source: Front Mol Biosci. 2025 Aug 22;12:1621357. doi: 10.3389/fmolb.2025.1621357 (PMC12411887; doi:10.3389/fmolb.2025.1621357)
Supplement: Supplementary file 1 [file Supplementaryfile1.docx]

**SUPPLEMENTARY INFORMATION**

## Supplementary Tables

**Supplementary Table 1.** Sample information

| **SampleID** | **sample_type** | **type** | **time** |
| --- | --- | --- | --- |
| PNC | Sample | LO | T0 |
| PNE | Labeled | LO | T0 |
| I_Palsa_C1 | Sample | PO | T1 |
| I_Palsa_C2 | Sample | PO | T1 |
| I_Palsa_N1 | Sample | PL | T1 |
| I_Palsa_N2 | Sample | PL | T1 |
| I_Palsa_E1 | Labeled | PL | T1 |
| I_Palsa_E2 | Labeled | PL | T1 |
| II_Palsa_C1 | Sample | PO | T2 |
| II_Palsa_C2 | Sample | PO | T2 |
| II_Palsa_N1 | Sample | PL | T2 |
| II_Palsa_N2 | Sample | PL | T2 |
| II_Palsa_E1 | Labeled | PL | T2 |
| II_Palsa_E2 | Labeled | PL | T2 |
| III_Palsa_C1 | Sample | PO | T3 |
| III_Palsa_C2 | Sample | PO | T3 |
| III_Palsa_N1 | Sample | PL | T3 |
| III_Palsa_N2 | Sample | PL | T3 |
| III_Palsa_E1 | Labeled | PL | T3 |
| III_Palsa_E2 | Labeled | PL | T3 |
| Palsa_T0 | Sample | PO | T0 |

**Supplementary Table 2.** Abundances of metabolites detected via UPLC-HRMS

**Supplementary Table 3.** Labeled status of metabolites detected via UPLC-HRMS

**Supplementary Table 4.** Annotation of metabolites detected via UPLC-HRMS

**Supplementary Table 5.** Abundances of metabolites detected via NMR

**Supplementary Table 6.** Labeled status of metabolites via NMR

**Supplementary Table 7.** Discriminant features between PL and PO samples determined via multiblock PLS-DA

##

## Supplementary Figures


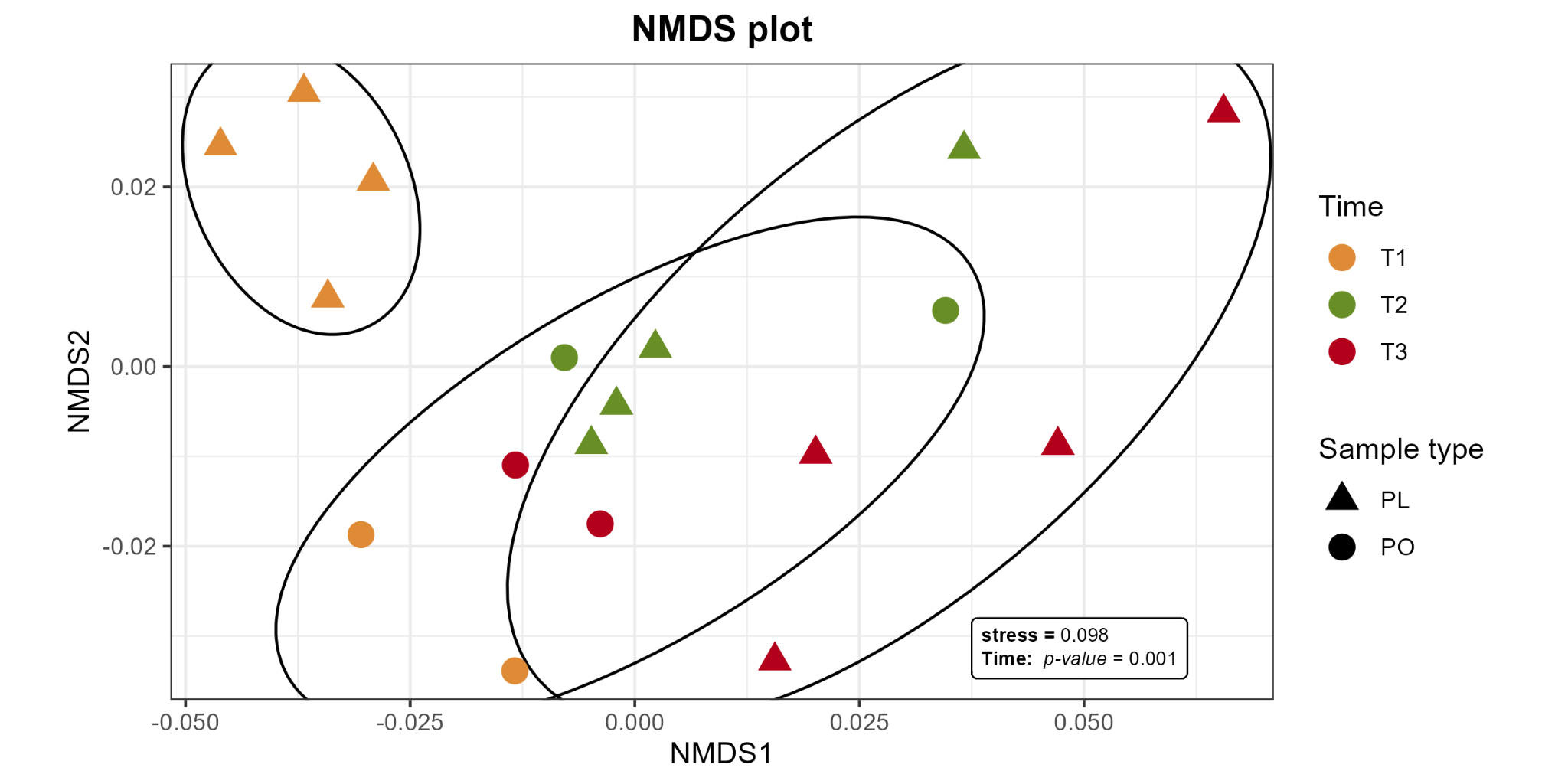


**Supplementary Figure 1.** Non-metric multidimensional scaling (NMDS) ordination of amended and unamended peat litters using Manhattan distances. Amended samples at T1, cluster separately from samples at any other time point (p-value = 0.001).


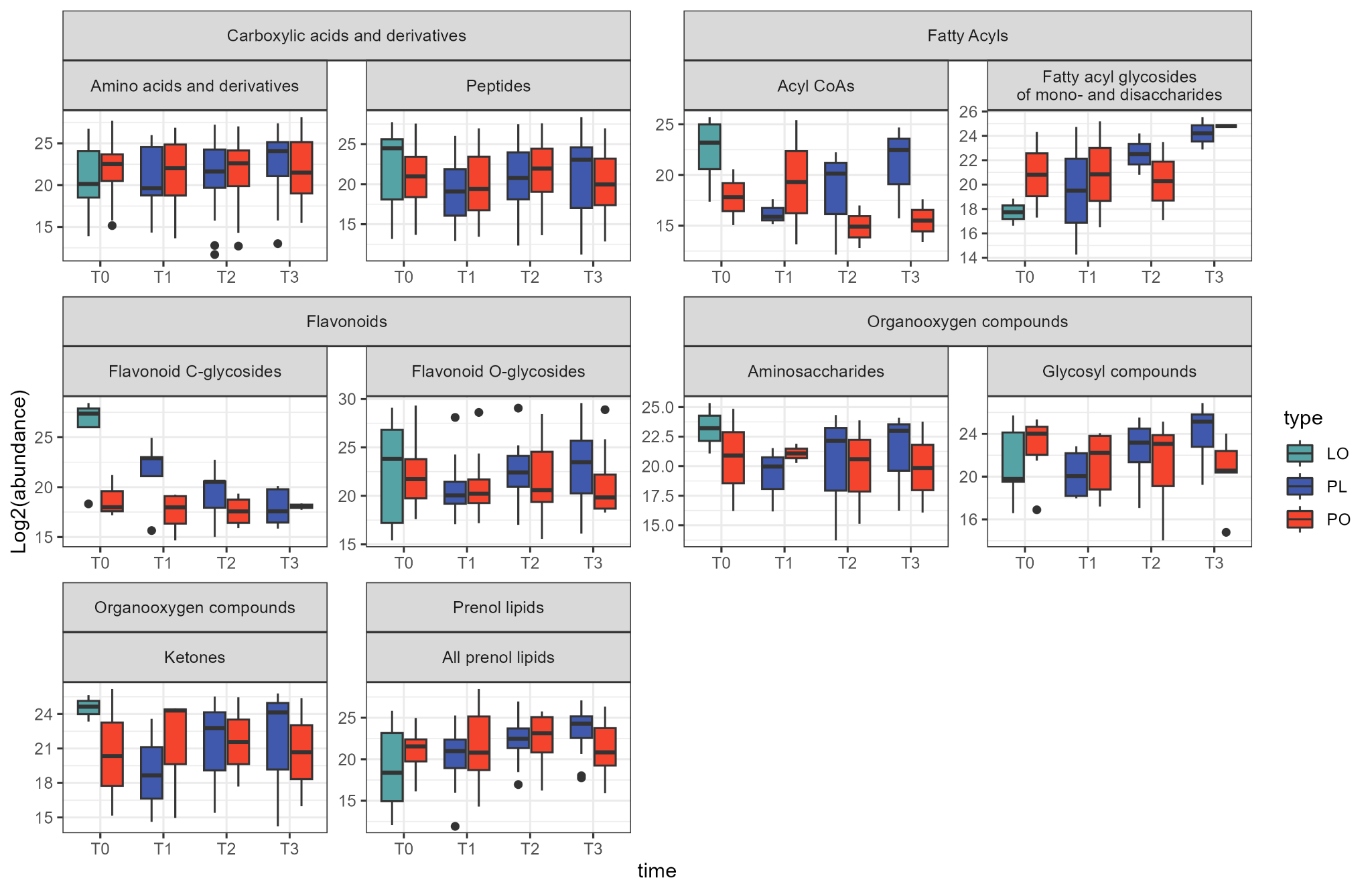


**Supplementary Figure 2.** Changes in abundance of different classes and subclasses of metabolites between different sample types


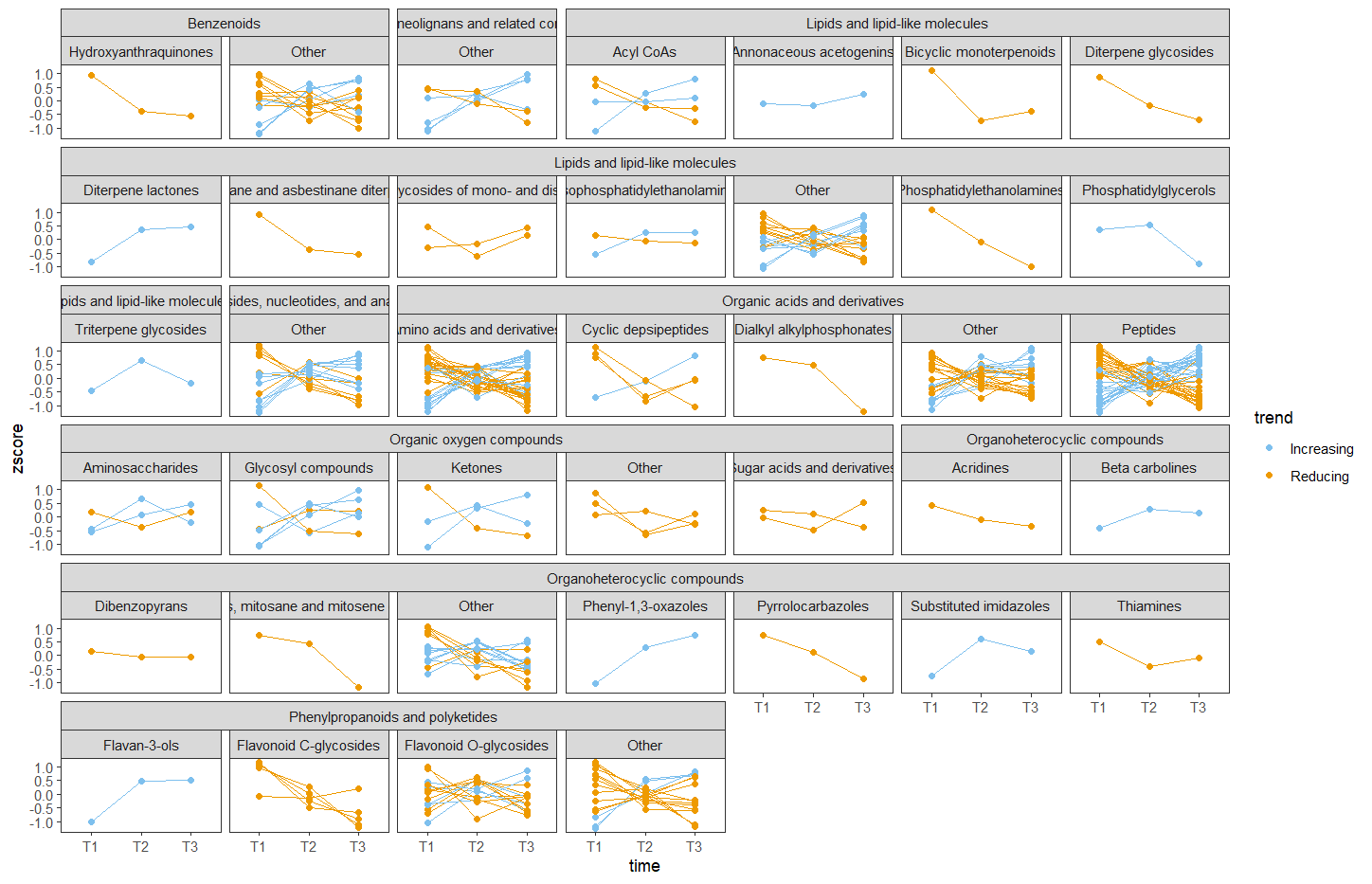


**Supplementary Figure 3.** Changes in abundance (expressed as z-score) of metabolites from different classes in the amended peat samples (PL) between T1 to T3.


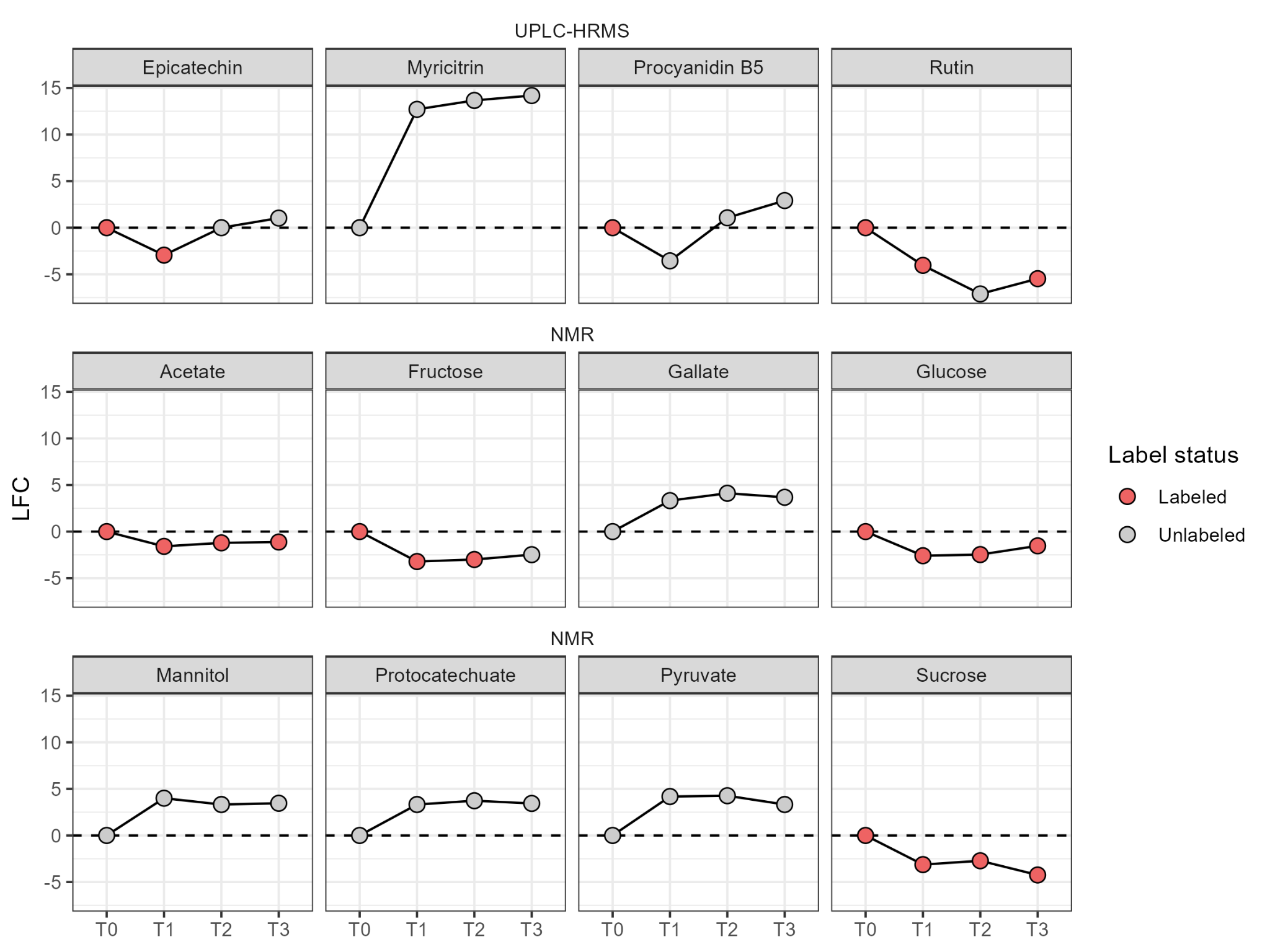


**Supplementary Figure 4.** Changes in abundance (expressed as log2 fold-change in relationship with unamended peat at T0) of litter derived flavonoids and some of their degradation products.


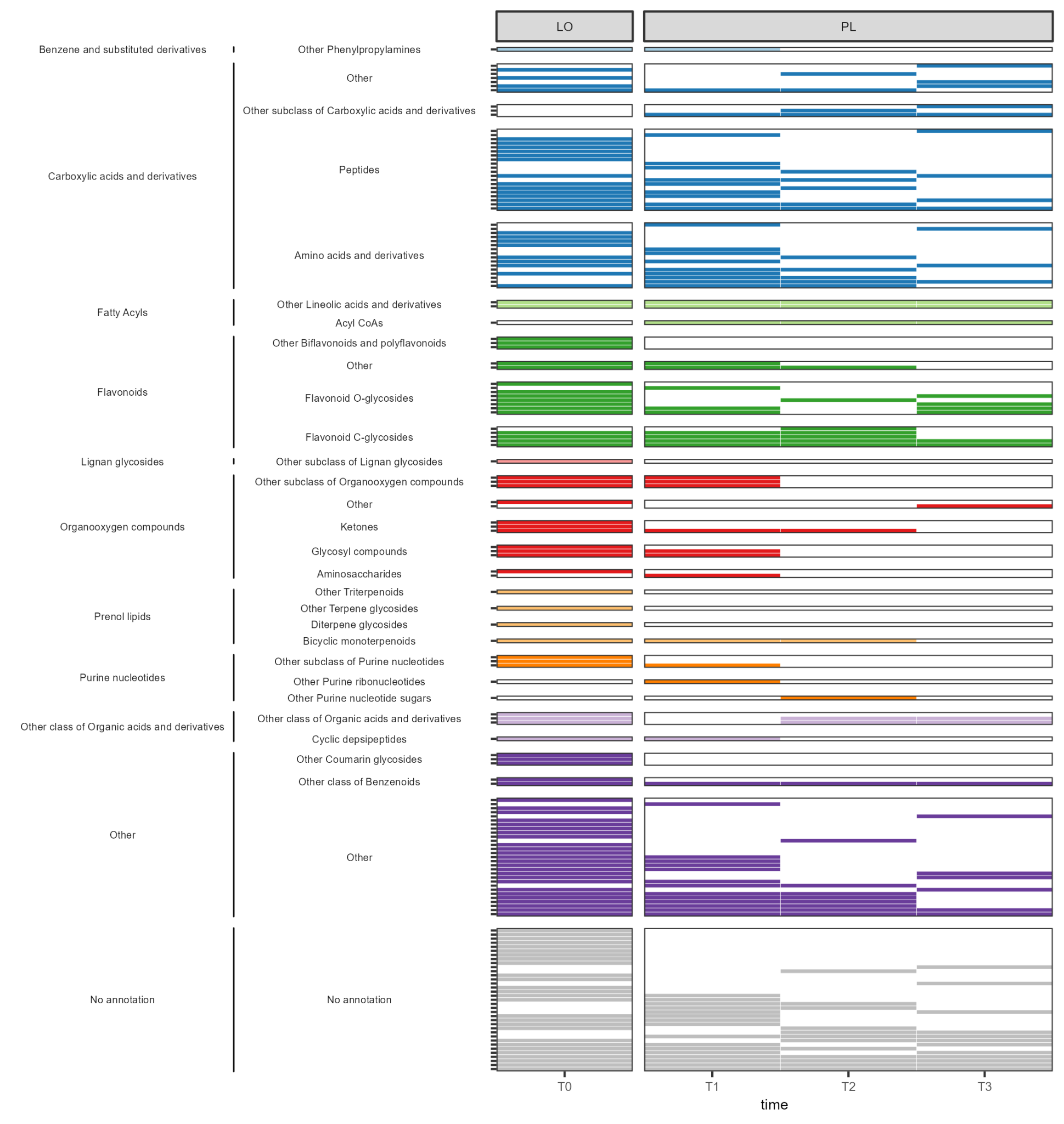


**Supplementary Figure 5.** Presence of labeled metabolites at different time points throughout the incubation in LO and PL samples.
